# Supplementary figures and images for: Cost-effectiveness evaluation of different control strategies for Clonorchis sinensis infection in a high endemic area of China: A modelling study
Source: PLoS Negl Trop Dis. 2022 May 23;16(5):e0010429. doi: 10.1371/journal.pntd.0010429 (PMC9166357; doi:10.1371/journal.pntd.0010429)

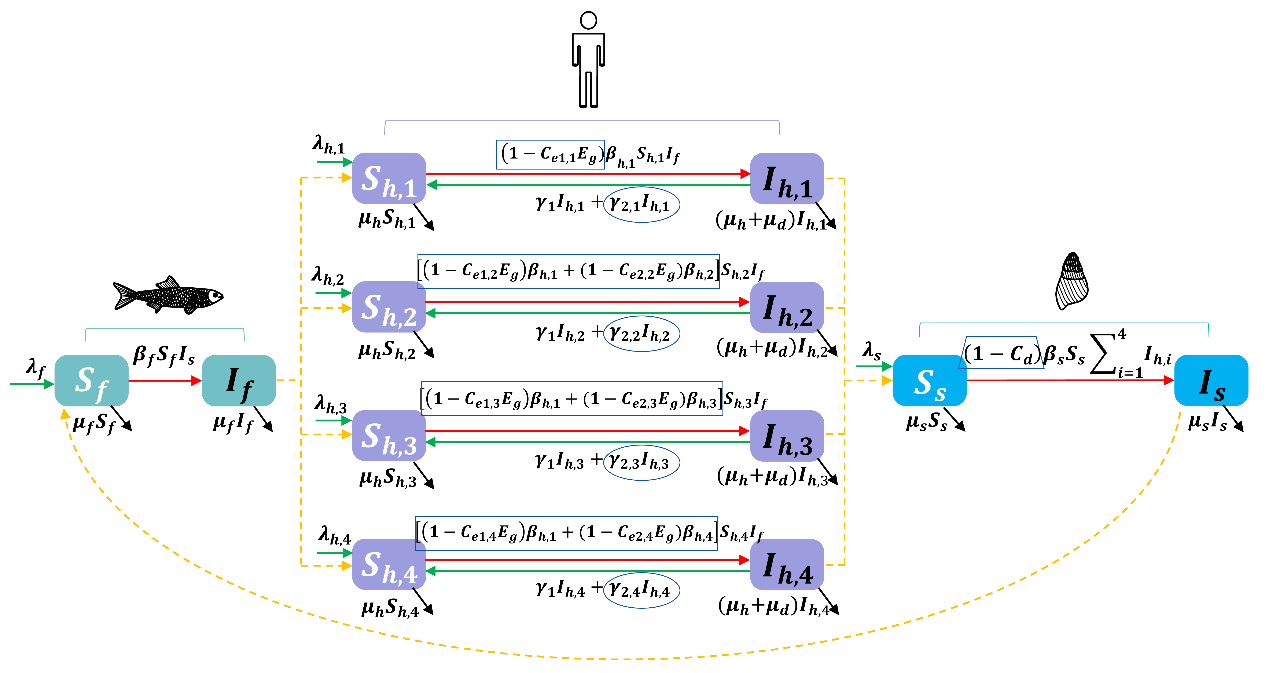

Supplement: S1 Fig — (TIF) [file pntd.0010429.s001.tif]
