# Supplementary material for: Cost-effectiveness evaluation of different control strategies for Clonorchis sinensis infection in a high endemic area of China: A modelling study
Source: PLoS Negl Trop Dis. 2022 May 23;16(5):e0010429. doi: 10.1371/journal.pntd.0010429 (PMC9166357; doi:10.1371/journal.pntd.0010429)
Supplement: S1 Table — (DOCX) [file pntd.0010429.s002.docx]

**S1 Table** **Descriptions of model parameters (unit: day^-1^).**

| Parameter | Description |
| --- | --- |
| $\lambda_{h,1}$ | Recruitment of susceptible humans who seldom eat raw or uncooked fish |
| $\lambda_{h,2}$ | Recruitment of susceptible humans who moderately eat raw or uncooked fish |
| $\lambda_{h,3}$ | Recruitment of susceptible humans who often eat raw or uncooked fish |
| $\lambda_{h,4}$ | Recruitment of susceptible humans who very often eat raw or uncooked fish |
| $\lambda_{s}$ | Recruitment of susceptible snails |
| $\lambda_{f}$ | Recruitment of susceptible fish |
| $\beta_{h,1}$ | Transmission rate from an infected fish to a susceptible human who seldom consumes raw fish |
| $c_{2}$ | Ratio of transmission rate from an infected fish to a susceptible human who moderately consumes raw or uncooked fish to that who seldom |
| $c_{3}$ | Ratio of transmission rate from an infected fish to a susceptible human who often consumes raw or uncooked fish to that who seldom |
| $c_{4}$ | Ratio of transmission rate from an infected fish to a susceptible human who very often consumes raw or uncooked fish to that who seldom |
| $\beta_{h,2}$ | The increased transmission rate from an infected fish to a susceptible human who moderately consumes raw fish, compared to that who seldom, which is equal to ${\beta_{h,1}(c}_{2}-1)$ |
| $\beta_{h,3}$ | The increased transmission rate from an infected fish to a susceptible human who often consumes raw fish, compared to that who seldom, which is equal to ${\beta_{h,1}(c}_{3}-1)$ |
| $\beta_{h,4}$ | The increased transmission rate from an infected fish to a susceptible human who very often consumes raw fish, compared to that who seldom, which is equal to ${\beta_{h,1}(c}_{4}-1)$ |
| $\beta_{s}$ | Transmission rate from an infected human to a susceptible snail |
| $\beta_{f}$ | Transmission rate from an infected snail to a susceptible fish |
| $\mu_{h}$ | Nature birth and death rate of human hosts |
| $\mu_{d}$ | Fatality rate from *C.sinensis* infections |
| $\mu_{s}$ | Birth and death rates of snails |
| $\mu_{f}$ | Birth and death rates of fish |
| $\gamma_{1}$ | Basic recovery rate of infected humans through individual treatment |
