# Supplementary material for: Cost-effectiveness evaluation of different control strategies for Clonorchis sinensis infection in a high endemic area of China: A modelling study
Source: PLoS Negl Trop Dis. 2022 May 23;16(5):e0010429. doi: 10.1371/journal.pntd.0010429 (PMC9166357; doi:10.1371/journal.pntd.0010429)
Supplement: S2 Table — (DOCX) [file pntd.0010429.s003.docx]

**S2 Table.** **Observed prevalence of *C. sinensis* infection among groups of people with different frequencies of raw fish consumption in Fusha Town.**[1]

| Frequency of eating raw fish per year | No. of samples | No. of positive (%) |
| --- | --- | --- |
| Seldom | 802 | 121 (15.08) |
| <5 times | 215 | 126 (58.60) |
| 5-10 times | 123 | 98 (79.67) |
| >10 times | 60 | 59 (98.33) |
| Total | 1200 | 404 (33.67) |

**References**

1. Du S, Huang J, Li H. [Epidemiology of clonorchiasis in the towns near the Pearl River Delta] (author’s tranl). South China J Prev Med. 2015;41(3):273–5. Chinese.
