# Supplementary material for: Cost-effectiveness evaluation of different control strategies for Clonorchis sinensis infection in a high endemic area of China: A modelling study
Source: PLoS Negl Trop Dis. 2022 May 23;16(5):e0010429. doi: 10.1371/journal.pntd.0010429 (PMC9166357; doi:10.1371/journal.pntd.0010429)
Supplement: S3 Table — (DOCX) [file pntd.0010429.s004.docx]

**S3 Table.** **The prior distributions and posterior estimations of model parameters (unit: day^-1^).**

| Parameter | Prior | |  | Posterior | |
| --- | --- | --- | --- | --- | --- |
|  | Mode [Range] | Source |  | Best set [95% CI] | Source |
| $p_{1}$ | 0.67 [0.57-0.77] | [1] |  | 0.67 [0.65-0.69] | fitting |
| $p_{2}$ | 0.18 [0.08-0.28] | [1] |  | 0.18 [0.16-0.20] | fitting |
| $p_{3}$ | 0.103 [0.053-0.153] | [1] |  | 0.100 [0.088-0.118] | fitting |
| $p_{4}$ | 0.050 [0.040-0.060] | [1] |  | 0.050 [0.044-0.055] | fitting |
| $\lambda_{h1}$ | $p_{1}^{'}\times N_{h}\times\mu_{h}+{\mu_{d}\times I}_{h1}$* | |  | 0.58 [0.56-0.60] | fitting |
| $\lambda_{h2}$ | $p_{2}^{'}\times N_{h}\times\mu_{h}+{\mu_{d}\times I}_{h2}$ | |  | 0.15 [0.14-0.17] | fitting |
| $\lambda_{h3}$ | $p_{3}^{'}\times N_{h}\times\mu_{h}+{\mu_{d}\times I}_{h3}$ | |  | 0.087 [0.077-0.103] | fitting |
| $\lambda_{h4}$ | $p_{4}^{'}\times N_{h}\times\mu_{h}+{\mu_{d}\times I}_{h4}$ | |  | 0.044 [0.039-0.049] | fitting |
| $\lambda_{s}$ | - | - |  | 3729.1 | [2,3,4] |
| $\lambda_{f}$ | 2191.78 [219.18-4383.56] | [3,5,6] |  | 2598.6 [1277.7-3272.8] | fitting |
| $\beta_{h1}$ | 2.81×10^-10^ [2.81×10^-11^-5.63×10^-10^] | Solving equations |  | 3.01×10^-10^ [1.40×10^-10^-4.06×10^-10^] | fitting |
| $\beta_{s}$ | 2.71×10^-9^ [2.71×10^-10^-5.43×10^-9^] | Same as $\beta_{h1}$ |  | 2.07×10^-9^ [1.64×10^-9^-3.95×10^-9^] | fitting |
| $\beta_{f}$ | 1.87×10^-8^ [1.87×10^-9^-3.75×10^-8^] | Same as $\beta_{h1}$ |  | 2.13×10^-8^ [1.14×10^-8^-2.82×10^-8^] | fitting |
| $c_{2}$ | 8 [3-13] | $\frac{{I_{h,2}}/{S_{h,2}}}{{I_{h,1}}/{S_{h,1}}}$ |  | 8.13 [6.23-10.51] | fitting |
| $c_{3}$ | 22 [17-32] | $\frac{{I_{h,3}}/{S_{h,3}}}{{I_{h,1}}/{S_{h,1}}}$ |  | 23.29 [18.63-28.30] | fitting |
| $c_{4}$ | 332 [312-352] | $\frac{{I_{h,4}}/{S_{h,4}}}{{I_{h,1}}/{S_{h,1}}}$ |  | 332.30 [320.52-346.53] | fitting |
| $\mu_{h}$ | - | - |  | 1.49×10^-5^ | [7] |
| $\mu_{d}$ | 2.10×10-7 [1.38×10-7-3.12×10-7] | [8,9,10] |  | 2.18×10^-7^ [1.65×10^-7^-2.86×10^-7^] | fitting |
| $\mu_{s}$ | - | - |  | 1/365 | [4] |
| $\mu_{f}$ | 1/(1.5×365) [1/(3×365)- 1/(1×365)] | [5] |  | 1/(1.62×365) [1/(2.06×365)-1/(1.15×365)] | fitting |
| $\gamma_{1}$ | 0.140/365 [0.014/365-0.200/365] | [11] |  | 0.172/365 [0.068/365-0.173/365)] | fitting |

*$p_{g}^{'} (g=1,2,3,4)$ represents adjusted proportion of $p_{g}$, to make the sum of $p_{g}^{'}$ for all groups equals to one.

**References**

1. Du S, Huang J, Li H. [Epidemiology of clonorchiasis in the towns near the Pearl River Delta] (author’s tranl). South China J Prev Med. 2015;41(3):273–5. Chinese.
2. Wang J, Zhou J, Lyu W, Zhang H, Li S, Zheng X, et al. [Effect of aquatic plant coverage and water depth on the diversity of large aquatic animals: A study based on ponds in the lower reaches of the Yangtze River]. Acta Agriculturae Shanghai. 2017;33(3):65-70. Chinese.
3. Xie X [Internet]. [The rent of fish pond in Zhongshan, Guangdong Province is up to 4750 yuan per mu, which breaking the record] (author's tranl). [cited 2021 Oct 27]. Available from: http://www.bbwfish.com/article.asp?artid=86551. Chinese.
4. Chen Z, Zou L, Shen D, Zhang W, Ruan S. Mathematical modelling and control of Schistosomiasis in Hubei Province, China. Acta Trop. 2010;115(1-2):119-25. <https://doi.org/10.1016/j.actatropica.2010.02.012>
5. Liu J, Zhang J, Yuan H. [The investigation and analysis about cost and benefit of freshwater fish breeding]. Chinese Fisheries Economics. 2017;35(1):18-27. Chinese.
6. Baiduzhidao [Internet]. [How many fish can be raised in one mu of fishpond?] (author's tranl). [cited 2021 Oct 27]. Available from: https://zhidao.baidu.com/question/1430988985257871219.html. Chinese.
7. Zhongshan Statistical Bureau [Internet]. [Zhongshan Statistical Yearbook 2013]. [cited 2021 Oct 27]. Available from: http://stats.zs.gov.cn/tjzl/tjnj/2013nj/index.htm. Chinese.
8. Fürst T, Keiser J, Utzinger J. Global burden of human food-borne trematodiasis: a systematic review and meta-analysis. Lancet Infect Dis. 2012;12(3):210-21. https://doi.org/10.1016/S1473-3099(11)70294-8
9. Parkin DM. The global health burden of infection-associated cancers in the year 2002. Int J Cancer. 2006;118(12):3030-44. https://doi.org/10.1002/ijc.21731
10. Khan AS, Dageforde LA. Cholangiocarcinoma. Surg Clin North Am. 2019;99(2):315-35. <https://doi.org/10.1016/j.suc.2018.12.004>
11. Ma J, Chen G, Tang X, Zuo Z, Cao F, Gong P, et al. [Survey of life habits and health knowledge of the people in epidemic region of Clonorchiasis]. J Trop Med. 2008;8(8):858-60, 865. Chinese.
