# Supplementary material for: Cost-effectiveness evaluation of different control strategies for Clonorchis sinensis infection in a high endemic area of China: A modelling study
Source: PLoS Negl Trop Dis. 2022 May 23;16(5):e0010429. doi: 10.1371/journal.pntd.0010429 (PMC9166357; doi:10.1371/journal.pntd.0010429)
Supplement: S4 Table — (DOCX) [file pntd.0010429.s005.docx]

**S4 Table** **Values set for compliance of chemotherapy among different kinds of targeted population.**

| Strategy | | Targeted population | Base value | Range | Distribution | Effective coverage of treatment | Reference |
| --- | --- | --- | --- | --- | --- | --- | --- |
| Chemotherapy | | Whole (${A_{w1}}^{*}$) | 54.76% | Base±25% | Triangular | ${C_{m}}^{\dagger}{A_{w1}}^{*}$ | [1,2,3] |
|  | | At-risk (${A_{k1}}^{*}$) | 71.64% | Base±25% | Triangular | ${C_{m}}^{\dagger}{A_{k1}}^{*}$ | [1] |
|  | | Positive | 100% | - | - | ${C_{m}}^{\dagger}$ | [1] |
| The increased compliance of chemotherapy due to IEC$(A_{incre}$) | ${A_{incre}}^{*}$ | | 31.52% | Base±25% | Triangular | - | [3] |
| Chemotherapy + IEC | | Whole (${A_{w2}}^{*}$) | ${A_{w1}}^{*}(1+C_{e}{A_{incre}}^{*})$ | - | - | ${C_{m}}^{\dagger}{A_{w1}}^{*}(1+{C_{e}}^{\ddagger}{A_{incre}}^{*})$ | - |
|  | | At-risk (${A_{k2}}^{*}$) | ${A_{k1}}^{*}(1+C_{e}{A_{incre}}^{*})$ | - | - | ${C_{m}}^{\dagger}{A_{k1}}^{*}(1+{C_{e}}^{\ddagger}{A_{incre}}^{*})$ | - |
|  | | Positive | 100% | - | - | ${C_{m}}^{\dagger}$ | [1] |

^*^*A* indicates the compliance of chemotherapy.

^†^$C_{m}$ is the coverage of chemotherapy.

^‡^$C_{e}$ is the coverage of IEC.

**References**

1. Fang Y, Ruan C, Gao X, Tan Q, Chen R, Hao Y. [Research on chemotherapy measures in different Clonorchis sinensis infectiosity endemic areas]. Chin J Schisto Control. 2014;26(3):300-2. Chinese.
2. Tan Q, Yu Z, Ma J, Tang X, Gong F, Cao F, et al. [Evaluation of health education and chemotherapeutic treatment on the prevention of clonorchiasis]. J Trop Med. 2012;12(4):478-80. Chinese.
3. Hu G, Hu J, Song K, Lin D, Zhang J, Cao C, et al. The role of health education and health promotion in the control of schistosomiasis: experiences from a 12-year intervention study in the Poyang Lake area. Acta Trop. 2005;96(2-3):232-41. https://doi.org/10.1016/j.actatropica.2005.07.016
