# Supplementary material for: Cost-effectiveness evaluation of different control strategies for Clonorchis sinensis infection in a high endemic area of China: A modelling study
Source: PLoS Negl Trop Dis. 2022 May 23;16(5):e0010429. doi: 10.1371/journal.pntd.0010429 (PMC9166357; doi:10.1371/journal.pntd.0010429)
Supplement: S5 Table — (DOCX) [file pntd.0010429.s006.docx]

**S5 Table.** **Values of intervention parameters of the current recommended control strategies^*^.**

| Parameter^#^ | WHO | The Chinese government | The government of Guangdong Province |
| --- | --- | --- | --- |
| $C_{d}$ | 0 | 0.85 | 0.90 |
| $C_{e1,g}(g=1,2,3,4)$ | 0 | 0 | 0.90 |
| $C_{e2,g}(g=2,3,4)$ | 0 | 0 | 0.90 |
| $C_{m,1}$ | 1.00 | 0 | 0 |
| $C_{m,2}$ | 1.00 | 0.80 | 0.80 |
| $C_{m,3}$ | 1.00 | 0.80 | 0.80 |
| $C_{m,4}$ | 1.00 | 0.80 | 0.80 |
| *F* | 1 | 1 | 1 |
| *D* | 10 | 10 | 10 |

^*^The Chinese government do have a strategy through IEC to improve the awareness of key knowledge on clonorchiasis control among students by 95%. However, such improvement does not have a direct effect on the system and is difficult to address. Thus, we set $C_{e1,g}=0 (g=1,2,3,4)$ and $C_{e2,g}=0 (g=2,3,4)$.

^#^$C_{d}$ is the coverage of sanitation toilets, $C_{e1,g}$ is the proportions of people who have received information on improving hygiene habits, $C_{e2,g}$ is the proportions of people who have received information on stopping raw-fish-eating behavior and $C_{m, g}$ is the coverage of chemotherapy. $g=1,2,3,4$ represent human groups who seldom, moderately, often and very often consume raw fish, respectively. *F* indicates the frequency of chemotherapy per year and *D* indicates the intervention duration of chemotherapy.
