# Supplementary material for: Cost-effectiveness evaluation of different control strategies for Clonorchis sinensis infection in a high endemic area of China: A modelling study
Source: PLoS Negl Trop Dis. 2022 May 23;16(5):e0010429. doi: 10.1371/journal.pntd.0010429 (PMC9166357; doi:10.1371/journal.pntd.0010429)
Supplement: S6 Table — (DOCX) [file pntd.0010429.s007.docx]

**S6 Table** **Costs of each aspect** **of the control strategies^*^.**

| Item | Aspect | Description | Base cost per unit (2020 US I$) | Ranges | Distribution | Reference |
| --- | --- | --- | --- | --- | --- | --- |
| **Chemotherapy** | Drug | Praziquantel（75mg/kg） | 8.12 | - | - | Market research |
|  |  | Albendazole（3.2g） | 2.39 | - | - | Market research |
|  | Delivery | Costs related to chemotherapy excluding drug costs | 0.51 | Base ± 25% | Triangular | [1] |
|  | Cost to identify infected individuals | Stool examination (Kato-Katz) | 5.15 | Base ± 25% | Triangular | [1] |
| **IEC** | - | Information education, group meetings, IEC materials and media, etc | 1.12 | Base ± 25% | Triangular | [2] |
| **Environmental modification** | Initial investment cost | Removal of toilets (per toilet) | 97.94 | Base ± 25% | Triangular | [3] |
|  | Recurrent cost | Construction of septic tank toilets (per toilet) | 452.65 | Base ± 25% | Triangular | [4] |
|  |  | Maintenance of toilets (per toilet per year) | 88.86 | Base ± 25% | Triangular | [5] |

^*^Costs were assumed to follow a triangular distribution, with the mode being the value extracted from the reference, and the upper and lower bounds being plus or minus 25% of the mode.

**References**

1. Qian M, Zhou C, Zhu H, Chen Y, Zhou X. Comparison on different treatment strategies against Clonorchis sinensis infection. Infect Dis Poverty. Forthcoming 2021.
2. Fang Y. [A study on the epidemiology and the control measurements of Clonorchis Sinensis in Guangdong Province] [dissertation]. Guangzhou: Sun Yat-sen University; 2006. Chinese
3. Baiduzhidao [Internet]. [How much does it cost to remove a toilet on a fish pond?] (author's tranl). [cited 2021 Oct 27]. Available from: https://zhidao.baidu.com/question/589637454610479365.html?entry=qb_uhome_tag. Chinese.
4. Miao Y, Yang Z, Zhou H. [Research on rural residents’ willingness to pay for environmental sanitation improvement and influencing factors: taking toilet improvement as an example] (author's tranl). Journal of Management World. 2012;9:89-99. Chinese
5. Huang S, Yu S, Ding H. [Investigation report on rural toilet renovation in Shandong Province] (author's tranl). Chinese Rural Science and Technology. 2018 Aug;279:75-9. Chinese.
