# Supplementary material for: Cost-effectiveness evaluation of different control strategies for Clonorchis sinensis infection in a high endemic area of China: A modelling study
Source: PLoS Negl Trop Dis. 2022 May 23;16(5):e0010429. doi: 10.1371/journal.pntd.0010429 (PMC9166357; doi:10.1371/journal.pntd.0010429)
Supplement: S7 Table — (DOCX) [file pntd.0010429.s008.docx]

**S7 Table. Simulation results under the effective control strategies that could reach infection control within 10 years^*^.**

| Targeted population | Strategy | | |  | Effectiveness | | |  | Total costs/US $ | |
| --- | --- | --- | --- | --- | --- | --- | --- | --- | --- | --- |
|  | $C_{d}$ | $C_{e}$ | $C_{m}$ |  | $R_{c}$ | $Y_{5\%}$ | Averted DALYs |  | Costs (PZQ) | Costs (ABZ) |
| Whole | 0.80 | 0.70 | 0.60 | 0.80 | | 8.01 | 12,596.6 | 3,785,599 | | 1,787,593 |
|  | 0.80 | 0.70 | 0.70 | 0.76 | | 7.01 | 12,708.7 | 4,287,254 | | 1,956,234 |
|  | 0.80 | 0.70 | 0.80 | 0.73 | | 7.01 | 12,803.0 | 4,788,911 | | 2,124,877 |
|  | 0.80 | 0.70 | 0.90 | 0.70 | | 6.01 | 12,884.2 | 5,290,570 | | 2,293,520 |
|  | 0.80 | 0.70 | 1.00 | 0.66 | | 6.01 | 12,955.5 | 5,792,231 | | 2,462,163 |
|  | 0.80 | 0.80 | 0.50 | 0.82 | | 9.01 | 12,578.6 | 3,349,091 | | 1,684,091 |
|  | 0.80 | 0.80 | 0.60 | 0.77 | | 8.01 | 12,704.2 | 3,850,744 | | 1,852,733 |
|  | 0.80 | 0.80 | 0.70 | 0.74 | | 7.01 | 12,807.1 | 4,352,400 | | 2,021,375 |
|  | 0.80 | 0.80 | 0.80 | 0.70 | | 6.01 | 12,893.6 | 4,854,058 | | 2,190,018 |
|  | 0.80 | 0.80 | 0.90 | 0.67 | | 6.01 | 12,968.0 | 5,355,719 | | 2,358,662 |
|  | 0.80 | 0.80 | 1.00 | 0.64 | | 6.01 | 13,033.3 | 5,857,381 | | 2,527,306 |
|  | 0.80 | 0.90 | 0.40 | 0.83 | | 9.01 | 12,553.1 | 2,912,584 | | 1,580,590 |
|  | 0.80 | 0.90 | 0.50 | 0.78 | | 8.01 | 12,697.5 | 3,414,235 | | 1,749,231 |
|  | 0.80 | 0.90 | 0.60 | 0.74 | | 7.01 | 12,811.8 | 3,915,889 | | 1,917,874 |
|  | 0.80 | 0.90 | 0.70 | 0.71 | | 6.01 | 12,905.2 | 4,417,546 | | 2,086,517 |
|  | 0.80 | 0.90 | 0.80 | 0.67 | | 6.01 | 12,983.7 | 4,919,206 | | 2,255,160 |
|  | 0.80 | 0.90 | 0.90 | 0.64 | | 6.01 | 13,051.0 | 5,420,867 | | 2,423,805 |
|  | 0.80 | 0.90 | 1.00 | 0.61 | | 5.01 | 13,110.1 | 5,922,531 | | 2,592,449 |
|  | 0.80 | 1.00 | 0.40 | 0.80 | | 9.01 | 12,686.4 | 2,977,727 | | 1,645,730 |
|  | 0.80 | 1.00 | 0.50 | 0.75 | | 7.01 | 12,816.4 | 3,479,379 | | 1,814,372 |
|  | 0.80 | 1.00 | 0.60 | 0.71 | | 7.01 | 12,919.1 | 3,981,035 | | 1,983,015 |
|  | 0.80 | 1.00 | 0.70 | 0.67 | | 6.01 | 13,002.7 | 4,482,693 | | 2,151,659 |
|  | 0.80 | 1.00 | 0.80 | 0.64 | | 6.01 | 13,072.7 | 4,984,354 | | 2,320,303 |
|  | 0.80 | 1.00 | 0.90 | 0.61 | | 5.01 | 13,132.5 | 5,486,017 | | 2,488,947 |
|  | 0.80 | 1.00 | 1.00 | 0.58 | | 5.01 | 13,184.9 | 5,987,681 | | 2,657,592 |
|  | 0.90 | 0.00 | 0.70 | 0.76 | | 9.01 | 12,594.3 | 3,871,224 | | 1,540,236 |
|  | 0.90 | 0.00 | 0.80 | 0.73 | | 8.01 | 12,684.8 | 4,372,872 | | 1,708,874 |
|  | 0.90 | 0.00 | 0.90 | 0.70 | | 8.01 | 12,763.2 | 4,874,523 | | 1,877,512 |
|  | 0.90 | 0.00 | 1.00 | 0.67 | | 7.01 | 12,832.2 | 5,376,176 | | 2,046,152 |
|  | 0.90 | 0.10 | 0.60 | 0.77 | | 9.01 | 12,567.8 | 3,434,722 | | 1,436,737 |
|  | 0.90 | 0.10 | 0.70 | 0.74 | | 8.01 | 12,668.9 | 3,936,368 | | 1,605,375 |
|  | 0.90 | 0.10 | 0.80 | 0.71 | | 8.01 | 12,754.6 | 4,438,018 | | 1,774,013 |
|  | 0.90 | 0.10 | 0.90 | 0.68 | | 7.01 | 12,828.8 | 4,939,670 | | 1,942,653 |
|  | 0.90 | 0.10 | 1.00 | 0.65 | | 7.01 | 12,894.1 | 5,441,324 | | 2,111,293 |
|  | 0.90 | 0.20 | 0.60 | 0.75 | | 9.01 | 12,646.2 | 3,499,864 | | 1,501,876 |
|  | 0.90 | 0.20 | 0.70 | 0.72 | | 8.01 | 12,741.6 | 4,001,512 | | 1,670,514 |
|  | 0.90 | 0.20 | 0.80 | 0.69 | | 8.01 | 12,822.4 | 4,503,163 | | 1,839,153 |
|  | 0.90 | 0.20 | 0.90 | 0.66 | | 7.01 | 12,892.2 | 5,004,816 | | 2,007,793 |
|  | 0.90 | 0.20 | 1.00 | 0.63 | | 7.01 | 12,953.8 | 5,506,472 | | 2,176,434 |
|  | 0.90 | 0.30 | 0.50 | 0.76 | | 9.01 | 12,614.4 | 3,063,362 | | 1,398,377 |
| Whole | 0.90 | 0.30 | 0.60 | 0.73 | | 8.01 | 12,722.7 | 3,565,007 | | 1,567,015 |
|  | 0.90 | 0.30 | 0.70 | 0.70 | | 8.01 | 12,812.2 | 4,066,657 | | 1,735,654 |
|  | 0.90 | 0.30 | 0.80 | 0.67 | | 7.01 | 12,888.0 | 4,568,309 | | 1,904,294 |
|  | 0.90 | 0.30 | 0.90 | 0.64 | | 7.01 | 12,953.5 | 5,069,963 | | 2,072,934 |
|  | 0.90 | 0.30 | 1.00 | 0.61 | | 6.01 | 13,011.1 | 5,571,620 | | 2,241,576 |
|  | 0.90 | 0.40 | 0.50 | 0.74 | | 9.01 | 12,695.6 | 3,128,503 | | 1,463,515 |
|  | 0.90 | 0.40 | 0.60 | 0.71 | | 8.01 | 12,796.8 | 3,630,151 | | 1,632,154 |
|  | 0.90 | 0.40 | 0.70 | 0.67 | | 7.01 | 12,880.4 | 4,131,801 | | 1,800,794 |
|  | 0.90 | 0.40 | 0.80 | 0.64 | | 7.01 | 12,951.1 | 4,633,455 | | 1,969,434 |
|  | 0.90 | 0.40 | 0.90 | 0.62 | | 7.01 | 13,012.1 | 5,135,110 | | 2,138,075 |
|  | 0.90 | 0.40 | 1.00 | 0.59 | | 6.01 | 13,065.9 | 5,636,768 | | 2,306,717 |
|  | 0.90 | 0.50 | 0.50 | 0.72 | | 9.01 | 12,774.2 | 3,193,646 | | 1,528,654 |
|  | 0.90 | 0.50 | 0.60 | 0.68 | | 8.01 | 12,868.4 | 3,695,294 | | 1,697,293 |
|  | 0.90 | 0.50 | 0.70 | 0.65 | | 7.01 | 12,945.9 | 4,196,946 | | 1,865,934 |
|  | 0.90 | 0.50 | 0.80 | 0.62 | | 7.01 | 13,011.3 | 4,698,601 | | 2,034,575 |
|  | 0.90 | 0.50 | 0.90 | 0.60 | | 6.01 | 13,067.8 | 5,200,258 | | 2,203,217 |
|  | 0.90 | 0.50 | 1.00 | 0.57 | | 6.01 | 13,117.4 | 5,701,917 | | 2,371,859 |
|  | 0.90 | 0.60 | 0.40 | 0.74 | | 9.01 | 12,740.8 | 2,757,142 | | 1,425,154 |
|  | 0.90 | 0.60 | 0.50 | 0.70 | | 8.01 | 12,849.7 | 3,258,788 | | 1,593,793 |
|  | 0.90 | 0.60 | 0.60 | 0.66 | | 7.01 | 12,936.6 | 3,760,438 | | 1,762,433 |
|  | 0.90 | 0.60 | 0.70 | 0.63 | | 7.01 | 13,007.9 | 4,262,091 | | 1,931,074 |
|  | 0.90 | 0.60 | 0.80 | 0.60 | | 6.01 | 13,068.0 | 4,763,747 | | 2,099,716 |
|  | 0.90 | 0.60 | 0.90 | 0.57 | | 6.01 | 13,119.8 | 5,265,405 | | 2,268,358 |
|  | 0.90 | 0.60 | 1.00 | 0.55 | | 6.01 | 13,165.4 | 5,767,065 | | 2,437,001 |
|  | 0.90 | 0.70 | 0.40 | 0.71 | | 9.01 | 12,821.6 | 2,822,283 | | 1,490,293 |
|  | 0.90 | 0.70 | 0.50 | 0.67 | | 8.01 | 12,921.6 | 3,323,931 | | 1,658,932 |
|  | 0.90 | 0.70 | 0.60 | 0.64 | | 7.01 | 13,001.0 | 3,825,582 | | 1,827,573 |
|  | 0.90 | 0.70 | 0.70 | 0.61 | | 7.01 | 13,066.0 | 4,327,237 | | 1,996,215 |
|  | 0.90 | 0.70 | 0.80 | 0.58 | | 6.01 | 13,120.7 | 4,828,894 | | 2,164,857 |
|  | 0.90 | 0.70 | 0.90 | 0.55 | | 6.01 | 13,167.7 | 5,330,553 | | 2,333,500 |
|  | 0.90 | 0.70 | 1.00 | 0.53 | | 5.01 | 13,209.2 | 5,832,214 | | 2,502,144 |
|  | 0.90 | 0.80 | 0.40 | 0.69 | | 8.01 | 12,898.1 | 2,887,424 | | 1,555,431 |
|  | 0.90 | 0.80 | 0.50 | 0.65 | | 7.01 | 12,989.1 | 3,389,074 | | 1,724,072 |
|  | 0.90 | 0.80 | 0.60 | 0.61 | | 7.01 | 13,060.9 | 3,890,727 | | 1,892,714 |
|  | 0.90 | 0.80 | 0.70 | 0.58 | | 6.01 | 13,119.6 | 4,392,383 | | 2,061,356 |
|  | 0.90 | 0.80 | 0.80 | 0.56 | | 6.01 | 13,168.8 | 4,894,041 | | 2,229,999 |
|  | 0.90 | 0.80 | 0.90 | 0.53 | | 5.01 | 13,211.3 | 5,395,702 | | 2,398,643 |
|  | 0.90 | 0.80 | 1.00 | 0.51 | | 5.01 | 13,248.8 | 5,897,364 | | 2,567,286 |
|  | 0.90 | 0.90 | 0.30 | 0.70 | | 9.01 | 12,862.0 | 2,450,920 | | 1,451,930 |
|  | 0.90 | 0.90 | 0.40 | 0.66 | | 8.01 | 12,969.9 | 2,952,566 | | 1,620,571 |
|  | 0.90 | 0.90 | 0.50 | 0.62 | | 7.01 | 13,051.7 | 3,454,217 | | 1,789,212 |
|  | 0.90 | 0.90 | 0.60 | 0.59 | | 6.01 | 13,116.1 | 3,955,872 | | 1,957,854 |
| Whole | 0.90 | 0.90 | 0.70 | 0.56 | | 6.01 | 13,168.5 | 4,457,529 | | 2,126,497 |
|  | 0.90 | 0.90 | 0.80 | 0.53 | | 6.01 | 13,212.5 | 4,959,189 | | 2,295,141 |
|  | 0.90 | 0.90 | 0.90 | 0.51 | | 5.01 | 13,250.5 | 5,460,850 | | 2,463,785 |
|  | 0.90 | 0.90 | 1.00 | 0.48 | | 5.01 | 13,284.2 | 5,962,513 | | 2,632,429 |
|  | 0.90 | 1.00 | 0.30 | 0.67 | | 9.01 | 12,940.0 | 2,516,060 | | 1,517,069 |
|  | 0.90 | 1.00 | 0.40 | 0.63 | | 8.01 | 13,036.6 | 3,017,709 | | 1,685,710 |
|  | 0.90 | 1.00 | 0.50 | 0.60 | | 7.01 | 13,109.3 | 3,519,361 | | 1,854,352 |
|  | 0.90 | 1.00 | 0.60 | 0.56 | | 6.01 | 13,166.4 | 4,021,017 | | 2,022,995 |
|  | 0.90 | 1.00 | 0.70 | 0.53 | | 6.01 | 13,212.8 | 4,522,676 | | 2,191,639 |
|  | 0.90 | 1.00 | 0.80 | 0.51 | | 5.01 | 13,251.9 | 5,024,336 | | 2,360,283 |
|  | 0.90 | 1.00 | 0.90 | 0.48 | | 5.01 | 13,285.8 | 5,525,999 | | 2,528,928 |
|  | 0.90 | 1.00 | 1.00 | 0.46 | | 5.01 | 13,316.0 | 6,027,663 | | 2,697,573 |
|  | 1.00 | 0.00 | 0.40 | 0.00 | | 9.01 | 12,939.9 | 2,406,284 | | 1,074,309 |
|  | 1.00 | 0.00 | 0.50 | 0.00 | | 8.01 | 12,987.3 | 2,907,923 | | 1,242,944 |
|  | 1.00 | 0.00 | 0.60 | 0.00 | | 8.01 | 13,028.5 | 3,409,565 | | 1,411,580 |
|  | 1.00 | 0.00 | 0.70 | 0.00 | | 7.01 | 13,065.3 | 3,911,210 | | 1,580,217 |
|  | 1.00 | 0.00 | 0.80 | 0.00 | | 7.01 | 13,098.5 | 4,412,859 | | 1,748,855 |
|  | 1.00 | 0.00 | 0.90 | 0.00 | | 7.01 | 13,129.2 | 4,914,510 | | 1,917,494 |
|  | 1.00 | 0.00 | 1.00 | 0.00 | | 6.01 | 13,157.7 | 5,416,163 | | 2,086,134 |
|  | 1.00 | 0.10 | 0.30 | 0.00 | | 9.92 | 12,910.2 | 1,969,789 | | 970,812 |
|  | 1.00 | 0.10 | 0.40 | 0.00 | | 9.01 | 12,964.8 | 2,471,424 | | 1,139,446 |
|  | 1.00 | 0.10 | 0.50 | 0.00 | | 8.01 | 13,011.2 | 2,973,063 | | 1,308,081 |
|  | 1.00 | 0.10 | 0.60 | 0.00 | | 7.01 | 13,051.6 | 3,474,707 | | 1,476,718 |
|  | 1.00 | 0.10 | 0.70 | 0.00 | | 7.01 | 13,087.6 | 3,976,354 | | 1,645,356 |
|  | 1.00 | 0.10 | 0.80 | 0.00 | | 7.01 | 13,120.2 | 4,478,004 | | 1,813,995 |
|  | 1.00 | 0.10 | 0.90 | 0.00 | | 6.01 | 13,150.3 | 4,979,656 | | 1,982,634 |
|  | 1.00 | 0.10 | 1.00 | 0.00 | | 6.01 | 13,178.3 | 5,481,310 | | 2,151,275 |
|  | 1.00 | 0.20 | 0.30 | 0.00 | | 9.45 | 12,936.1 | 2,034,927 | | 1,035,949 |
|  | 1.00 | 0.20 | 0.40 | 0.00 | | 8.80 | 12,989.6 | 2,536,563 | | 1,204,583 |
|  | 1.00 | 0.20 | 0.50 | 0.00 | | 8.01 | 13,035.0 | 3,038,204 | | 1,373,219 |
|  | 1.00 | 0.20 | 0.60 | 0.00 | | 7.01 | 13,074.6 | 3,539,849 | | 1,541,857 |
|  | 1.00 | 0.20 | 0.70 | 0.00 | | 7.01 | 13,109.8 | 4,041,498 | | 1,710,495 |
|  | 1.00 | 0.20 | 0.80 | 0.00 | | 7.01 | 13,141.7 | 4,543,149 | | 1,879,135 |
|  | 1.00 | 0.20 | 0.90 | 0.00 | | 6.01 | 13,171.1 | 5,044,802 | | 2,047,775 |
|  | 1.00 | 0.20 | 1.00 | 0.00 | | 6.01 | 13,198.6 | 5,546,458 | | 2,216,416 |
|  | 1.00 | 0.30 | 0.30 | 0.00 | | 9.01 | 12,962.1 | 2,100,065 | | 1,101,085 |
|  | 1.00 | 0.30 | 0.40 | 0.00 | | 8.01 | 13,014.5 | 2,601,703 | | 1,269,721 |
|  | 1.00 | 0.30 | 0.50 | 0.00 | | 8.01 | 13,058.8 | 3,103,346 | | 1,438,358 |
|  | 1.00 | 0.30 | 0.60 | 0.00 | | 7.01 | 13,097.4 | 3,604,992 | | 1,606,996 |
|  | 1.00 | 0.30 | 0.70 | 0.00 | | 7.01 | 13,131.7 | 4,106,642 | | 1,775,635 |
|  | 1.00 | 0.30 | 0.80 | 0.00 | | 6.01 | 13,162.9 | 4,608,294 | | 1,944,275 |
|  | 1.00 | 0.30 | 0.90 | 0.00 | | 6.01 | 13,191.7 | 5,109,949 | | 2,112,916 |
| Whole | 1.00 | 0.30 | 1.00 | 0.00 | | 6.01 | 13,218.6 | 5,611,605 | | 2,281,557 |
|  | 1.00 | 0.40 | 0.30 | 0.00 | | 9.01 | 12,988.3 | 2,165,204 | | 1,166,222 |
|  | 1.00 | 0.40 | 0.40 | 0.00 | | 8.01 | 13,039.4 | 2,666,843 | | 1,334,858 |
|  | 1.00 | 0.40 | 0.50 | 0.00 | | 7.01 | 13,082.6 | 3,168,487 | | 1,503,496 |
|  | 1.00 | 0.40 | 0.60 | 0.00 | | 7.01 | 13,120.1 | 3,670,135 | | 1,672,135 |
|  | 1.00 | 0.40 | 0.70 | 0.00 | | 7.01 | 13,153.5 | 4,171,786 | | 1,840,775 |
|  | 1.00 | 0.40 | 0.80 | 0.00 | | 6.01 | 13,183.9 | 4,673,440 | | 2,009,415 |
|  | 1.00 | 0.40 | 0.90 | 0.00 | | 6.01 | 13,211.9 | 5,175,095 | | 2,178,057 |
|  | 1.00 | 0.40 | 1.00 | 0.00 | | 6.01 | 13,238.3 | 5,676,753 | | 2,346,699 |
|  | 1.00 | 0.50 | 0.30 | 0.00 | | 9.01 | 13,014.8 | 2,230,343 | | 1,231,359 |
|  | 1.00 | 0.50 | 0.40 | 0.00 | | 8.01 | 13,064.4 | 2,731,984 | | 1,399,996 |
|  | 1.00 | 0.50 | 0.50 | 0.00 | | 7.01 | 13,106.3 | 3,233,629 | | 1,568,635 |
|  | 1.00 | 0.50 | 0.60 | 0.00 | | 7.01 | 13,142.8 | 3,735,278 | | 1,737,274 |
|  | 1.00 | 0.50 | 0.70 | 0.00 | | 6.01 | 13,175.2 | 4,236,931 | | 1,905,915 |
|  | 1.00 | 0.50 | 0.80 | 0.00 | | 6.01 | 13,204.7 | 4,738,585 | | 2,074,556 |
|  | 1.00 | 0.50 | 0.90 | 0.00 | | 6.01 | 13,232.0 | 5,240,242 | | 2,243,198 |
|  | 1.00 | 0.50 | 1.00 | 0.00 | | 5.01 | 13,257.7 | 5,741,901 | | 2,411,840 |
|  | 1.00 | 0.60 | 0.30 | 0.00 | | 8.96 | 13,041.5 | 2,295,482 | | 1,296,497 |
|  | 1.00 | 0.60 | 0.40 | 0.00 | | 8.01 | 13,089.6 | 2,797,125 | | 1,465,135 |
|  | 1.00 | 0.60 | 0.50 | 0.00 | | 7.01 | 13,130.2 | 3,298,772 | | 1,633,774 |
|  | 1.00 | 0.60 | 0.60 | 0.00 | | 7.01 | 13,165.5 | 3,800,422 | | 1,802,414 |
|  | 1.00 | 0.60 | 0.70 | 0.00 | | 6.01 | 13,196.9 | 4,302,075 | | 1,971,055 |
|  | 1.00 | 0.60 | 0.80 | 0.00 | | 6.01 | 13,225.5 | 4,803,731 | | 2,139,697 |
|  | 1.00 | 0.60 | 0.90 | 0.00 | | 6.01 | 13,251.9 | 5,305,389 | | 2,308,339 |
|  | 1.00 | 0.60 | 1.00 | 0.00 | | 5.01 | 13,276.8 | 5,807,049 | | 2,476,982 |
|  | 1.00 | 0.70 | 0.20 | 0.00 | | 9.85 | 13,011.4 | 1,858,983 | | 1,192,997 |
|  | 1.00 | 0.70 | 0.30 | 0.00 | | 8.30 | 13,068.6 | 2,360,622 | | 1,361,635 |
|  | 1.00 | 0.70 | 0.40 | 0.00 | | 7.68 | 13,115.1 | 2,862,266 | | 1,530,273 |
|  | 1.00 | 0.70 | 0.50 | 0.00 | | 7.01 | 13,154.2 | 3,363,914 | | 1,698,913 |
|  | 1.00 | 0.70 | 0.60 | 0.00 | | 6.01 | 13,188.2 | 3,865,566 | | 1,867,554 |
|  | 1.00 | 0.70 | 0.70 | 0.00 | | 6.01 | 13,218.5 | 4,367,221 | | 2,036,196 |
|  | 1.00 | 0.70 | 0.80 | 0.00 | | 6.01 | 13,246.1 | 4,868,878 | | 2,204,838 |
|  | 1.00 | 0.70 | 0.90 | 0.00 | | 5.01 | 13,271.7 | 5,370,537 | | 2,373,481 |
|  | 1.00 | 0.70 | 1.00 | 0.00 | | 5.01 | 13,295.8 | 5,872,198 | | 2,542,125 |
|  | 1.00 | 0.80 | 0.20 | 0.00 | | 9.41 | 13,040.9 | 1,924,121 | | 1,258,134 |
|  | 1.00 | 0.80 | 0.30 | 0.00 | | 8.01 | 13,096.1 | 2,425,762 | | 1,426,772 |
|  | 1.00 | 0.80 | 0.40 | 0.00 | | 7.01 | 13,140.8 | 2,927,407 | | 1,595,412 |
|  | 1.00 | 0.80 | 0.50 | 0.00 | | 7.01 | 13,178.4 | 3,429,057 | | 1,764,053 |
|  | 1.00 | 0.80 | 0.60 | 0.00 | | 6.01 | 13,211.1 | 3,930,710 | | 1,932,694 |
|  | 1.00 | 0.80 | 0.70 | 0.00 | | 6.01 | 13,240.2 | 4,432,366 | | 2,101,337 |
|  | 1.00 | 0.80 | 0.80 | 0.00 | | 5.01 | 13,266.7 | 4,934,024 | | 2,269,980 |
|  | 1.00 | 0.80 | 0.90 | 0.00 | | 5.01 | 13,291.3 | 5,435,685 | | 2,438,623 |
| Whole | 1.00 | 0.80 | 1.00 | 0.00 | | 5.01 | 13,314.7 | 5,937,347 | | 2,607,267 |
|  | 1.00 | 0.90 | 0.20 | 0.00 | | 9.01 | 13,071.2 | 1,989,260 | | 1,323,272 |
|  | 1.00 | 0.90 | 0.30 | 0.00 | | 8.01 | 13,124.2 | 2,490,902 | | 1,491,911 |
|  | 1.00 | 0.90 | 0.40 | 0.00 | | 7.01 | 13,167.0 | 2,992,549 | | 1,660,551 |
|  | 1.00 | 0.90 | 0.50 | 0.00 | | 6.01 | 13,202.9 | 3,494,200 | | 1,829,193 |
|  | 1.00 | 0.90 | 0.60 | 0.00 | | 6.01 | 13,234.2 | 3,995,855 | | 1,997,835 |
|  | 1.00 | 0.90 | 0.70 | 0.00 | | 5.01 | 13,262.0 | 4,497,512 | | 2,166,478 |
|  | 1.00 | 0.90 | 0.80 | 0.00 | | 5.01 | 13,287.4 | 4,999,171 | | 2,335,122 |
|  | 1.00 | 0.90 | 0.90 | 0.00 | | 5.01 | 13,311.0 | 5,500,833 | | 2,503,766 |
|  | 1.00 | 0.90 | 1.00 | 0.00 | | 5.01 | 13,333.4 | 6,002,496 | | 2,672,410 |
|  | 1.00 | 1.00 | 0.20 | 0.00 | | 9.01 | 13,102.3 | 2,054,399 | | 1,388,409 |
|  | 1.00 | 1.00 | 0.30 | 0.00 | | 8.01 | 13,152.9 | 2,556,042 | | 1,557,049 |
|  | 1.00 | 1.00 | 0.40 | 0.00 | | 7.01 | 13,193.7 | 3,057,691 | | 1,725,691 |
|  | 1.00 | 1.00 | 0.50 | 0.00 | | 6.01 | 13,227.9 | 3,559,344 | | 1,894,333 |
|  | 1.00 | 1.00 | 0.60 | 0.00 | | 6.01 | 13,257.5 | 4,061,000 | | 2,062,976 |
|  | 1.00 | 1.00 | 0.70 | 0.00 | | 5.01 | 13,284.0 | 4,562,658 | | 2,231,620 |
|  | 1.00 | 1.00 | 0.80 | 0.00 | | 5.01 | 13,308.1 | 5,064,319 | | 2,400,264 |
|  | 1.00 | 1.00 | 0.90 | 0.00 | | 5.01 | 13,330.7 | 5,565,981 | | 2,568,908 |
|  | 1.00 | 1.00 | 1.00 | 0.00 | | 4.01 | 13,352.1 | 6,067,645 | | 2,737,553 |
| At-risk | 0.80 | 1.00 | 0.90 | 0.57 | | 9.01 | 12,979.3 | 2,444,489 | | 1,466,476 |
|  | 0.80 | 1.00 | 1.00 | 0.53 | | 9.01 | 13,038.0 | 2,608,201 | | 1,521,512 |
|  | 0.90 | 0.60 | 1.00 | 0.51 | | 9.01 | 13,008.4 | 2,387,600 | | 1,300,929 |
|  | 0.90 | 0.70 | 0.80 | 0.55 | | 9.01 | 12,971.9 | 2,125,328 | | 1,256,000 |
|  | 0.90 | 0.70 | 0.90 | 0.52 | | 9.01 | 13,019.3 | 2,289,036 | | 1,311,035 |
|  | 0.90 | 0.70 | 1.00 | 0.49 | | 9.01 | 13,064.7 | 2,452,746 | | 1,366,069 |
|  | 0.90 | 0.80 | 0.70 | 0.55 | | 9.01 | 12,983.6 | 2,026,764 | | 1,266,106 |
|  | 0.90 | 0.80 | 0.80 | 0.52 | | 9.01 | 13,031.3 | 2,190,472 | | 1,321,140 |
|  | 0.90 | 0.80 | 0.90 | 0.50 | | 9.01 | 13,075.4 | 2,354,181 | | 1,376,175 |
|  | 0.90 | 0.80 | 1.00 | 0.47 | | 8.01 | 13,118.1 | 2,517,892 | | 1,431,210 |
|  | 0.90 | 0.90 | 0.60 | 0.56 | | 9.01 | 12,994.8 | 1,928,200 | | 1,276,211 |
|  | 0.90 | 0.90 | 0.70 | 0.53 | | 9.01 | 13,043.8 | 2,091,907 | | 1,331,246 |
|  | 0.90 | 0.90 | 0.80 | 0.50 | | 9.01 | 13,087.5 | 2,255,616 | | 1,386,281 |
|  | 0.90 | 0.90 | 0.90 | 0.47 | | 8.01 | 13,128.2 | 2,419,327 | | 1,441,316 |
|  | 0.90 | 0.90 | 1.00 | 0.45 | | 8.01 | 13,168.3 | 2,583,038 | | 1,496,351 |
|  | 0.90 | 1.00 | 0.50 | 0.57 | | 9.01 | 13,004.7 | 1,829,637 | | 1,286,316 |
|  | 0.90 | 1.00 | 0.60 | 0.53 | | 9.01 | 13,056.2 | 1,993,343 | | 1,341,351 |
|  | 0.90 | 1.00 | 0.70 | 0.50 | | 9.01 | 13,100.5 | 2,157,052 | | 1,396,386 |
|  | 0.90 | 1.00 | 0.80 | 0.48 | | 8.01 | 13,140.3 | 2,320,761 | | 1,451,421 |
|  | 0.90 | 1.00 | 0.90 | 0.45 | | 8.01 | 13,177.7 | 2,484,472 | | 1,506,457 |
|  | 0.90 | 1.00 | 1.00 | 0.42 | | 8.01 | 13,215.3 | 2,648,185 | | 1,561,493 |
|  | 1.00 | 0.00 | 0.60 | 0.00 | | 9.87 | 12,925.9 | 1,381,914 | | 729,948 |
|  | 1.00 | 0.00 | 0.70 | 0.00 | | 9.01 | 12,956.9 | 1,545,613 | | 784,979 |
| At-risk | 1.00 | 0.00 | 0.80 | 0.00 | | 9.01 | 12,985.9 | 1,709,314 | | 840,010 |
|  | 1.00 | 0.00 | 0.90 | 0.00 | | 9.01 | 13,013.7 | 1,873,017 | | 895,041 |
|  | 1.00 | 0.00 | 1.00 | 0.00 | | 9.01 | 13,040.7 | 2,036,721 | | 950,074 |
|  | 1.00 | 0.10 | 0.60 | 0.00 | | 9.44 | 12,953.0 | 1,447,054 | | 795,086 |
|  | 1.00 | 0.10 | 0.70 | 0.00 | | 9.01 | 12,983.7 | 1,610,754 | | 850,116 |
|  | 1.00 | 0.10 | 0.80 | 0.00 | | 9.01 | 13,012.6 | 1,774,456 | | 905,148 |
|  | 1.00 | 0.10 | 0.90 | 0.00 | | 9.01 | 13,040.2 | 1,938,159 | | 960,180 |
|  | 1.00 | 0.10 | 1.00 | 0.00 | | 9.01 | 13,067.2 | 2,101,865 | | 1,015,212 |
|  | 1.00 | 0.20 | 0.50 | 0.00 | | 9.85 | 12,947.3 | 1,348,496 | | 805,192 |
|  | 1.00 | 0.20 | 0.60 | 0.00 | | 9.01 | 12,980.2 | 1,512,194 | | 860,223 |
|  | 1.00 | 0.20 | 0.70 | 0.00 | | 9.01 | 13,010.6 | 1,675,895 | | 915,254 |
|  | 1.00 | 0.20 | 0.80 | 0.00 | | 9.01 | 13,039.1 | 1,839,598 | | 970,286 |
|  | 1.00 | 0.20 | 0.90 | 0.00 | | 9.01 | 13,066.6 | 2,003,302 | | 1,025,319 |
|  | 1.00 | 0.20 | 1.00 | 0.00 | | 8.01 | 13,093.5 | 2,167,008 | | 1,080,352 |
|  | 1.00 | 0.30 | 0.50 | 0.00 | | 9.43 | 12,975.0 | 1,413,635 | | 870,330 |
|  | 1.00 | 0.30 | 0.60 | 0.00 | | 9.01 | 13,007.4 | 1,577,335 | | 925,361 |
|  | 1.00 | 0.30 | 0.70 | 0.00 | | 9.01 | 13,037.3 | 1,741,037 | | 980,392 |
|  | 1.00 | 0.30 | 0.80 | 0.00 | | 9.01 | 13,065.6 | 1,904,740 | | 1,035,425 |
|  | 1.00 | 0.30 | 0.90 | 0.00 | | 8.01 | 13,092.8 | 2,068,446 | | 1,090,458 |
|  | 1.00 | 0.30 | 1.00 | 0.00 | | 8.01 | 13,119.6 | 2,232,152 | | 1,145,491 |
|  | 1.00 | 0.40 | 0.40 | 0.00 | | 9.98 | 12,967.3 | 1,315,077 | | 880,436 |
|  | 1.00 | 0.40 | 0.50 | 0.00 | | 9.01 | 13,002.7 | 1,478,775 | | 935,467 |
|  | 1.00 | 0.40 | 0.60 | 0.00 | | 9.01 | 13,034.6 | 1,642,475 | | 990,499 |
|  | 1.00 | 0.40 | 0.70 | 0.00 | | 9.01 | 13,064.1 | 1,806,178 | | 1,045,531 |
|  | 1.00 | 0.40 | 0.80 | 0.00 | | 8.39 | 13,091.9 | 1,969,883 | | 1,100,564 |
|  | 1.00 | 0.40 | 0.90 | 0.00 | | 8.01 | 13,118.8 | 2,133,589 | | 1,155,597 |
|  | 1.00 | 0.40 | 1.00 | 0.00 | | 8.01 | 13,145.6 | 2,297,297 | | 1,210,631 |
|  | 1.00 | 0.50 | 0.40 | 0.00 | | 9.59 | 12,996.0 | 1,380,216 | | 945,573 |
|  | 1.00 | 0.50 | 0.50 | 0.00 | | 9.01 | 13,030.7 | 1,543,915 | | 1,000,605 |
|  | 1.00 | 0.50 | 0.60 | 0.00 | | 9.01 | 13,061.9 | 1,707,616 | | 1,055,637 |
|  | 1.00 | 0.50 | 0.70 | 0.00 | | 8.61 | 13,090.8 | 1,871,320 | | 1,110,669 |
|  | 1.00 | 0.50 | 0.80 | 0.00 | | 8.01 | 13,118.2 | 2,035,026 | | 1,165,703 |
|  | 1.00 | 0.50 | 0.90 | 0.00 | | 8.01 | 13,144.8 | 2,198,733 | | 1,220,736 |
|  | 1.00 | 0.50 | 1.00 | 0.00 | | 8.01 | 13,171.4 | 2,362,441 | | 1,275,770 |
|  | 1.00 | 0.60 | 0.40 | 0.00 | | 9.15 | 13,024.9 | 1,445,355 | | 1,010,711 |
|  | 1.00 | 0.60 | 0.50 | 0.00 | | 9.01 | 13,058.8 | 1,609,055 | | 1,065,743 |
|  | 1.00 | 0.60 | 0.60 | 0.00 | | 8.83 | 13,089.3 | 1,772,758 | | 1,120,775 |
|  | 1.00 | 0.60 | 0.70 | 0.00 | | 8.01 | 13,117.6 | 1,936,462 | | 1,175,808 |
|  | 1.00 | 0.60 | 0.80 | 0.00 | | 8.01 | 13,144.4 | 2,100,169 | | 1,230,842 |
|  | 1.00 | 0.60 | 0.90 | 0.00 | | 8.01 | 13,170.6 | 2,263,877 | | 1,285,876 |
|  | 1.00 | 0.60 | 1.00 | 0.00 | | 8.01 | 13,197.0 | 2,427,586 | | 1,340,910 |
|  | 1.00 | 0.70 | 0.30 | 0.00 | | 9.95 | 13,016.3 | 1,346,796 | | 1,020,817 |
| At-risk | 1.00 | 0.70 | 0.40 | 0.00 | | 9.01 | 13,054.1 | 1,510,494 | | 1,075,848 |
|  | 1.00 | 0.70 | 0.50 | 0.00 | | 9.01 | 13,087.1 | 1,674,196 | | 1,130,881 |
|  | 1.00 | 0.70 | 0.60 | 0.00 | | 8.05 | 13,116.8 | 1,837,899 | | 1,185,914 |
|  | 1.00 | 0.70 | 0.70 | 0.00 | | 8.01 | 13,144.4 | 2,001,605 | | 1,240,948 |
|  | 1.00 | 0.70 | 0.80 | 0.00 | | 8.01 | 13,170.6 | 2,165,312 | | 1,295,982 |
|  | 1.00 | 0.70 | 0.90 | 0.00 | | 8.01 | 13,196.3 | 2,329,021 | | 1,351,016 |
|  | 1.00 | 0.70 | 1.00 | 0.00 | | 8.01 | 13,222.5 | 2,492,731 | | 1,406,051 |
|  | 1.00 | 0.80 | 0.30 | 0.00 | | 9.56 | 13,047.1 | 1,411,935 | | 1,085,954 |
|  | 1.00 | 0.80 | 0.40 | 0.00 | | 9.01 | 13,083.8 | 1,575,634 | | 1,140,986 |
|  | 1.00 | 0.80 | 0.50 | 0.00 | | 8.59 | 13,115.7 | 1,739,337 | | 1,196,019 |
|  | 1.00 | 0.80 | 0.60 | 0.00 | | 8.01 | 13,144.5 | 1,903,041 | | 1,251,053 |
|  | 1.00 | 0.80 | 0.70 | 0.00 | | 8.01 | 13,171.3 | 2,066,748 | | 1,306,087 |
|  | 1.00 | 0.80 | 0.80 | 0.00 | | 8.01 | 13,196.8 | 2,230,456 | | 1,361,121 |
|  | 1.00 | 0.80 | 0.90 | 0.00 | | 8.01 | 13,222.0 | 2,394,166 | | 1,416,156 |
|  | 1.00 | 0.80 | 1.00 | 0.00 | | 7.01 | 13,248.0 | 2,557,876 | | 1,471,191 |
|  | 1.00 | 0.90 | 0.30 | 0.00 | | 9.12 | 13,078.5 | 1,477,074 | | 1,151,091 |
|  | 1.00 | 0.90 | 0.40 | 0.00 | | 9.01 | 13,113.9 | 1,640,774 | | 1,206,124 |
|  | 1.00 | 0.90 | 0.50 | 0.00 | | 8.01 | 13,144.7 | 1,804,478 | | 1,261,158 |
|  | 1.00 | 0.90 | 0.60 | 0.00 | | 8.01 | 13,172.5 | 1,968,184 | | 1,316,192 |
|  | 1.00 | 0.90 | 0.70 | 0.00 | | 8.01 | 13,198.3 | 2,131,891 | | 1,371,227 |
|  | 1.00 | 0.90 | 0.80 | 0.00 | | 7.87 | 13,223.1 | 2,295,600 | | 1,426,262 |
|  | 1.00 | 0.90 | 0.90 | 0.00 | | 7.01 | 13,247.6 | 2,459,311 | | 1,481,297 |
|  | 1.00 | 0.90 | 1.00 | 0.00 | | 7.01 | 13,273.4 | 2,623,022 | | 1,536,332 |
|  | 1.00 | 1.00 | 0.30 | 0.00 | | 9.01 | 13,110.6 | 1,542,213 | | 1,216,229 |
|  | 1.00 | 1.00 | 0.40 | 0.00 | | 8.53 | 13,144.6 | 1,705,915 | | 1,271,263 |
|  | 1.00 | 1.00 | 0.50 | 0.00 | | 8.01 | 13,174.1 | 1,869,620 | | 1,326,297 |
|  | 1.00 | 1.00 | 0.60 | 0.00 | | 8.01 | 13,200.8 | 2,033,326 | | 1,381,332 |
|  | 1.00 | 1.00 | 0.70 | 0.00 | | 7.73 | 13,225.6 | 2,197,035 | | 1,436,367 |
|  | 1.00 | 1.00 | 0.80 | 0.00 | | 7.01 | 13,249.4 | 2,360,745 | | 1,491,402 |
|  | 1.00 | 1.00 | 0.90 | 0.00 | | 7.01 | 13,273.3 | 2,524,456 | | 1,546,438 |
|  | 1.00 | 1.00 | 1.00 | 0.00 | | 7.01 | 13,298.7 | 2,688,168 | | 1,601,474 |
| Positive | 0.80 | 0.70 | 0.60 | 0.80 | | 8.01 | 12,597.4 | 3,003,337 | | 2,714,860 |
|  | 0.80 | 0.70 | 0.70 | 0.76 | | 7.01 | 12,709.5 | 3,340,331 | | 3,026,529 |
|  | 0.80 | 0.70 | 0.80 | 0.73 | | 7.01 | 12,803.8 | 3,673,027 | | 3,336,753 |
|  | 0.80 | 0.70 | 0.90 | 0.70 | | 6.01 | 12,884.9 | 4,002,327 | | 3,645,836 |
|  | 0.80 | 0.70 | 1.00 | 0.66 | | 6.01 | 12,956.2 | 4,328,894 | | 3,954,002 |
|  | 0.80 | 0.80 | 0.50 | 0.82 | | 9.01 | 12,561.5 | 2,715,632 | | 2,463,004 |
|  | 0.80 | 0.80 | 0.60 | 0.78 | | 8.01 | 12,687.6 | 3,056,525 | | 2,775,983 |
|  | 0.80 | 0.80 | 0.70 | 0.74 | | 7.01 | 12,791.0 | 3,392,009 | | 3,087,143 |
|  | 0.80 | 0.80 | 0.80 | 0.71 | | 6.01 | 12,878.0 | 3,723,305 | | 3,396,898 |
|  | 0.80 | 0.80 | 0.90 | 0.67 | | 6.01 | 12,952.7 | 4,051,304 | | 3,705,544 |
|  | 0.80 | 0.80 | 1.00 | 0.64 | | 6.01 | 13,018.3 | 4,376,659 | | 4,013,302 |
| Positive | 0.80 | 0.90 | 0.50 | 0.79 | | 8.01 | 12,665.9 | 2,770,064 | | 2,524,544 |
|  | 0.80 | 0.90 | 0.60 | 0.75 | | 7.01 | 12,781.3 | 3,109,257 | | 2,836,952 |
|  | 0.80 | 0.90 | 0.70 | 0.72 | | 7.01 | 12,875.7 | 3,443,172 | | 3,147,586 |
|  | 0.80 | 0.90 | 0.80 | 0.68 | | 6.01 | 12,955.0 | 3,773,016 | | 3,456,852 |
|  | 0.80 | 0.90 | 0.90 | 0.65 | | 6.01 | 13,023.1 | 4,099,667 | | 3,765,046 |
|  | 0.80 | 0.90 | 1.00 | 0.62 | | 5.01 | 13,082.6 | 4,423,767 | | 4,072,383 |
|  | 0.80 | 1.00 | 0.40 | 0.81 | | 9.01 | 12,642.8 | 2,479,810 | | 2,271,832 |
|  | 0.80 | 1.00 | 0.50 | 0.77 | | 8.01 | 12,774.0 | 2,824,043 | | 2,585,933 |
|  | 0.80 | 1.00 | 0.60 | 0.73 | | 7.01 | 12,878.1 | 3,161,463 | | 2,897,744 |
|  | 0.80 | 1.00 | 0.70 | 0.69 | | 6.01 | 12,963.2 | 3,493,742 | | 3,207,829 |
|  | 0.80 | 1.00 | 0.80 | 0.66 | | 6.01 | 13,034.4 | 3,822,074 | | 3,516,587 |
|  | 0.80 | 1.00 | 0.90 | 0.63 | | 5.01 | 13,095.4 | 4,147,322 | | 3,824,310 |
|  | 0.80 | 1.00 | 1.00 | 0.60 | | 5.01 | 13,148.5 | 4,470,115 | | 4,131,207 |
|  | 0.90 | 0.00 | 0.60 | 0.75 | | 9.01 | 12,625.9 | 2,636,303 | | 2,315,326 |
|  | 0.90 | 0.00 | 0.70 | 0.71 | | 8.01 | 12,729.6 | 2,980,717 | | 2,629,486 |
|  | 0.90 | 0.00 | 0.80 | 0.68 | | 7.01 | 12,817.5 | 3,320,654 | | 2,942,142 |
|  | 0.90 | 0.00 | 0.90 | 0.65 | | 7.01 | 12,893.8 | 3,657,026 | | 3,253,600 |
|  | 0.90 | 0.00 | 1.00 | 0.62 | | 7.01 | 12,961.5 | 3,990,505 | | 3,564,088 |
|  | 0.90 | 0.10 | 0.50 | 0.77 | | 9.01 | 12,559.3 | 2,343,743 | | 2,061,840 |
|  | 0.90 | 0.10 | 0.60 | 0.73 | | 8.01 | 12,678.8 | 2,692,673 | | 2,377,517 |
|  | 0.90 | 0.10 | 0.70 | 0.70 | | 8.01 | 12,777.4 | 3,035,960 | | 2,691,298 |
|  | 0.90 | 0.10 | 0.80 | 0.67 | | 7.01 | 12,860.9 | 3,374,844 | | 3,003,601 |
|  | 0.90 | 0.10 | 0.90 | 0.63 | | 7.01 | 12,933.4 | 3,710,227 | | 3,314,727 |
|  | 0.90 | 0.10 | 1.00 | 0.61 | | 6.01 | 12,997.6 | 4,042,773 | | 3,624,901 |
|  | 0.90 | 0.20 | 0.50 | 0.76 | | 9.01 | 12,619.3 | 2,401,138 | | 2,124,375 |
|  | 0.90 | 0.20 | 0.60 | 0.72 | | 8.01 | 12,732.5 | 2,748,829 | | 2,439,636 |
|  | 0.90 | 0.20 | 0.70 | 0.68 | | 8.01 | 12,825.8 | 3,090,963 | | 2,753,030 |
|  | 0.90 | 0.20 | 0.80 | 0.65 | | 7.01 | 12,904.9 | 3,428,770 | | 3,064,971 |
|  | 0.90 | 0.20 | 0.90 | 0.62 | | 7.01 | 12,973.4 | 3,763,141 | | 3,375,757 |
|  | 0.90 | 0.20 | 1.00 | 0.59 | | 6.01 | 13,034.2 | 4,094,734 | | 3,685,611 |
|  | 0.90 | 0.30 | 0.50 | 0.74 | | 9.01 | 12,680.1 | 2,458,333 | | 2,186,843 |
|  | 0.90 | 0.30 | 0.60 | 0.70 | | 8.01 | 12,786.8 | 2,804,753 | | 2,501,677 |
|  | 0.90 | 0.30 | 0.70 | 0.67 | | 7.01 | 12,874.8 | 3,145,705 | | 2,814,674 |
|  | 0.90 | 0.30 | 0.80 | 0.64 | | 7.01 | 12,949.3 | 3,482,409 | | 3,126,245 |
|  | 0.90 | 0.30 | 0.90 | 0.61 | | 6.01 | 13,013.7 | 3,815,746 | | 3,436,684 |
|  | 0.90 | 0.30 | 1.00 | 0.58 | | 6.01 | 13,070.8 | 4,146,363 | | 3,746,210 |
|  | 0.90 | 0.40 | 0.50 | 0.73 | | 9.01 | 12,741.2 | 2,515,311 | | 2,249,238 |
|  | 0.90 | 0.40 | 0.60 | 0.69 | | 8.01 | 12,841.4 | 2,860,426 | | 2,563,634 |
|  | 0.90 | 0.40 | 0.70 | 0.66 | | 7.01 | 12,923.9 | 3,200,166 | | 2,876,224 |
|  | 0.90 | 0.40 | 0.80 | 0.63 | | 7.01 | 12,993.6 | 3,535,739 | | 3,187,414 |
|  | 0.90 | 0.40 | 0.90 | 0.60 | | 6.01 | 13,053.9 | 3,868,015 | | 3,497,497 |
|  | 0.90 | 0.40 | 1.00 | 0.57 | | 6.01 | 13,107.2 | 4,197,633 | | 3,806,688 |
| Positive | 0.90 | 0.50 | 0.40 | 0.75 | | 9.01 | 12,685.2 | 2,221,331 | | 1,995,274 |
|  | 0.90 | 0.50 | 0.50 | 0.71 | | 8.01 | 12,802.4 | 2,572,053 | | 2,311,554 |
|  | 0.90 | 0.50 | 0.60 | 0.67 | | 8.01 | 12,895.8 | 2,915,826 | | 2,625,499 |
|  | 0.90 | 0.50 | 0.70 | 0.64 | | 7.01 | 12,972.6 | 3,254,321 | | 2,937,671 |
|  | 0.90 | 0.50 | 0.80 | 0.61 | | 7.01 | 13,037.4 | 3,588,730 | | 3,248,471 |
|  | 0.90 | 0.50 | 0.90 | 0.58 | | 6.01 | 13,093.4 | 3,919,917 | | 3,558,188 |
|  | 0.90 | 0.50 | 1.00 | 0.56 | | 6.01 | 13,142.8 | 4,248,509 | | 3,867,034 |
|  | 0.90 | 0.60 | 0.40 | 0.73 | | 9.01 | 12,754.2 | 2,279,308 | | 2,058,005 |
|  | 0.90 | 0.60 | 0.50 | 0.69 | | 8.01 | 12,862.9 | 2,628,537 | | 2,373,783 |
|  | 0.90 | 0.60 | 0.60 | 0.66 | | 7.01 | 12,949.4 | 2,970,927 | | 2,687,264 |
|  | 0.90 | 0.60 | 0.70 | 0.62 | | 7.01 | 13,020.3 | 3,308,138 | | 2,999,005 |
|  | 0.90 | 0.60 | 0.80 | 0.59 | | 6.01 | 13,080.1 | 3,641,350 | | 3,309,403 |
|  | 0.90 | 0.60 | 0.90 | 0.57 | | 6.01 | 13,131.6 | 3,971,416 | | 3,618,743 |
|  | 0.90 | 0.60 | 1.00 | 0.54 | | 6.01 | 13,177.1 | 4,298,953 | | 3,927,234 |
|  | 0.90 | 0.70 | 0.40 | 0.71 | | 9.01 | 12,822.1 | 2,337,049 | | 2,120,657 |
|  | 0.90 | 0.70 | 0.50 | 0.67 | | 8.01 | 12,922.1 | 2,684,734 | | 2,435,917 |
|  | 0.90 | 0.70 | 0.60 | 0.64 | | 7.01 | 13,001.5 | 3,025,696 | | 2,748,918 |
|  | 0.90 | 0.70 | 0.70 | 0.61 | | 7.01 | 13,066.5 | 3,361,581 | | 3,060,213 |
|  | 0.90 | 0.70 | 0.80 | 0.58 | | 6.01 | 13,121.1 | 3,693,558 | | 3,370,196 |
|  | 0.90 | 0.70 | 0.90 | 0.55 | | 6.01 | 13,168.2 | 4,022,465 | | 3,679,147 |
|  | 0.90 | 0.70 | 1.00 | 0.53 | | 5.01 | 13,209.6 | 4,348,913 | | 3,987,273 |
|  | 0.90 | 0.80 | 0.40 | 0.69 | | 8.01 | 12,888.1 | 2,394,527 | | 2,183,220 |
|  | 0.90 | 0.80 | 0.50 | 0.65 | | 8.01 | 12,979.2 | 2,740,612 | | 2,497,943 |
|  | 0.90 | 0.80 | 0.60 | 0.62 | | 7.01 | 13,051.5 | 3,080,094 | | 2,810,447 |
|  | 0.90 | 0.80 | 0.70 | 0.59 | | 6.01 | 13,110.4 | 3,414,604 | | 3,121,281 |
|  | 0.90 | 0.80 | 0.80 | 0.56 | | 6.01 | 13,159.9 | 3,745,301 | | 3,430,833 |
|  | 0.90 | 0.80 | 0.90 | 0.54 | | 6.01 | 13,202.6 | 4,073,010 | | 3,739,382 |
|  | 0.90 | 0.80 | 1.00 | 0.51 | | 5.01 | 13,240.1 | 4,398,329 | | 4,047,128 |
|  | 0.90 | 0.90 | 0.30 | 0.71 | | 9.01 | 12,843.5 | 2,098,504 | | 1,928,570 |
|  | 0.90 | 0.90 | 0.40 | 0.67 | | 8.01 | 12,951.5 | 2,451,708 | | 2,245,684 |
|  | 0.90 | 0.90 | 0.50 | 0.63 | | 7.01 | 13,033.9 | 2,796,128 | | 2,559,847 |
|  | 0.90 | 0.90 | 0.60 | 0.60 | | 7.01 | 13,098.9 | 3,134,069 | | 2,871,834 |
|  | 0.90 | 0.90 | 0.70 | 0.57 | | 6.01 | 13,151.9 | 3,467,151 | | 3,182,188 |
|  | 0.90 | 0.90 | 0.80 | 0.54 | | 6.01 | 13,196.4 | 3,796,515 | | 3,491,293 |
|  | 0.90 | 0.90 | 0.90 | 0.52 | | 5.01 | 13,234.7 | 4,122,976 | | 3,799,423 |
|  | 0.90 | 0.90 | 1.00 | 0.49 | | 5.01 | 13,268.5 | 4,447,120 | | 4,106,774 |
|  | 0.90 | 1.00 | 0.30 | 0.68 | | 9.01 | 12,915.3 | 2,157,224 | | 1,991,550 |
|  | 0.90 | 1.00 | 0.40 | 0.64 | | 8.01 | 13,012.1 | 2,508,547 | | 2,308,033 |
|  | 0.90 | 1.00 | 0.50 | 0.61 | | 7.01 | 13,085.7 | 2,851,227 | | 2,621,612 |
|  | 0.90 | 1.00 | 0.60 | 0.58 | | 6.01 | 13,143.7 | 3,187,558 | | 2,933,057 |
|  | 0.90 | 1.00 | 0.70 | 0.55 | | 6.01 | 13,190.9 | 3,519,145 | | 3,242,909 |
|  | 0.90 | 1.00 | 0.80 | 0.52 | | 5.01 | 13,230.5 | 3,847,116 | | 3,551,546 |
| Positive | 0.90 | 1.00 | 0.90 | 0.50 | | 5.01 | 13,264.7 | 4,172,269 | | 3,859,237 |
|  | 0.90 | 1.00 | 1.00 | 0.48 | | 5.01 | 13,294.9 | 4,495,181 | | 4,166,175 |
|  | 1.00 | 0.00 | 0.30 | 0.00 | | 9.01 | 12,922.4 | 1,581,940 | | 1,392,296 |
|  | 1.00 | 0.00 | 0.40 | 0.00 | | 8.01 | 12,982.3 | 1,945,033 | | 1,712,731 |
|  | 1.00 | 0.00 | 0.50 | 0.00 | | 8.01 | 13,032.8 | 2,299,284 | | 2,030,195 |
|  | 1.00 | 0.00 | 0.60 | 0.00 | | 7.01 | 13,076.6 | 2,646,955 | | 2,345,448 |
|  | 1.00 | 0.00 | 0.70 | 0.00 | | 7.01 | 13,115.8 | 2,989,629 | | 2,659,023 |
|  | 1.00 | 0.00 | 0.80 | 0.00 | | 6.01 | 13,151.5 | 3,328,423 | | 2,971,295 |
|  | 1.00 | 0.00 | 0.90 | 0.00 | | 6.01 | 13,184.6 | 3,664,134 | | 3,282,532 |
|  | 1.00 | 0.00 | 1.00 | 0.00 | | 6.01 | 13,215.9 | 3,997,340 | | 3,592,927 |
|  | 1.00 | 0.10 | 0.30 | 0.00 | | 9.01 | 12,941.6 | 1,643,021 | | 1,456,069 |
|  | 1.00 | 0.10 | 0.40 | 0.00 | | 8.01 | 12,999.7 | 2,004,920 | | 1,776,103 |
|  | 1.00 | 0.10 | 0.50 | 0.00 | | 8.01 | 13,048.7 | 2,358,052 | | 2,093,191 |
|  | 1.00 | 0.10 | 0.60 | 0.00 | | 7.01 | 13,091.3 | 2,704,670 | | 2,408,091 |
|  | 1.00 | 0.10 | 0.70 | 0.00 | | 7.01 | 13,129.3 | 3,046,349 | | 2,721,332 |
|  | 1.00 | 0.10 | 0.80 | 0.00 | | 6.01 | 13,163.9 | 3,384,198 | | 3,033,287 |
|  | 1.00 | 0.10 | 0.90 | 0.00 | | 6.01 | 13,196.0 | 3,719,008 | | 3,344,221 |
|  | 1.00 | 0.10 | 1.00 | 0.00 | | 6.01 | 13,226.4 | 4,051,351 | | 3,654,326 |
|  | 1.00 | 0.20 | 0.30 | 0.00 | | 9.01 | 12,961.3 | 1,703,994 | | 1,519,806 |
|  | 1.00 | 0.20 | 0.40 | 0.00 | | 8.01 | 13,017.6 | 2,064,666 | | 1,839,428 |
|  | 1.00 | 0.20 | 0.50 | 0.00 | | 7.01 | 13,065.0 | 2,416,648 | | 2,156,130 |
|  | 1.00 | 0.20 | 0.60 | 0.00 | | 7.01 | 13,106.3 | 2,762,185 | | 2,470,667 |
|  | 1.00 | 0.20 | 0.70 | 0.00 | | 7.01 | 13,143.1 | 3,102,844 | | 2,783,566 |
|  | 1.00 | 0.20 | 0.80 | 0.00 | | 6.01 | 13,176.6 | 3,439,724 | | 3,095,195 |
|  | 1.00 | 0.20 | 0.90 | 0.00 | | 6.01 | 13,207.8 | 3,773,610 | | 3,405,818 |
|  | 1.00 | 0.20 | 1.00 | 0.00 | | 6.01 | 13,237.2 | 4,105,068 | | 3,715,626 |
|  | 1.00 | 0.30 | 0.30 | 0.00 | | 9.01 | 12,981.5 | 1,764,850 | | 1,583,504 |
|  | 1.00 | 0.30 | 0.40 | 0.00 | | 8.01 | 13,035.9 | 2,124,261 | | 1,902,702 |
|  | 1.00 | 0.30 | 0.50 | 0.00 | | 7.01 | 13,081.8 | 2,475,061 | | 2,219,007 |
|  | 1.00 | 0.30 | 0.60 | 0.00 | | 7.01 | 13,121.7 | 2,819,486 | | 2,533,171 |
|  | 1.00 | 0.30 | 0.70 | 0.00 | | 6.01 | 13,157.3 | 3,159,095 | | 2,845,717 |
|  | 1.00 | 0.30 | 0.80 | 0.00 | | 6.01 | 13,189.7 | 3,494,980 | | 3,157,012 |
|  | 1.00 | 0.30 | 0.90 | 0.00 | | 6.01 | 13,219.8 | 3,827,917 | | 3,467,317 |
|  | 1.00 | 0.30 | 1.00 | 0.00 | | 6.01 | 13,248.3 | 4,158,465 | | 3,776,820 |
|  | 1.00 | 0.40 | 0.30 | 0.00 | | 9.01 | 13,002.3 | 1,825,581 | | 1,647,160 |
|  | 1.00 | 0.40 | 0.40 | 0.00 | | 8.01 | 13,054.8 | 2,183,692 | | 1,965,922 |
|  | 1.00 | 0.40 | 0.50 | 0.00 | | 7.01 | 13,099.1 | 2,533,273 | | 2,281,818 |
|  | 1.00 | 0.40 | 0.60 | 0.00 | | 7.01 | 13,137.6 | 2,876,554 | | 2,595,597 |
|  | 1.00 | 0.40 | 0.70 | 0.00 | | 6.01 | 13,172.0 | 3,215,083 | | 2,907,780 |
|  | 1.00 | 0.40 | 0.80 | 0.00 | | 6.01 | 13,203.2 | 3,549,943 | | 3,218,731 |
|  | 1.00 | 0.40 | 0.90 | 0.00 | | 6.01 | 13,232.2 | 3,881,902 | | 3,528,708 |
|  | 1.00 | 0.40 | 1.00 | 0.00 | | 5.01 | 13,259.7 | 4,211,516 | | 3,837,896 |
| Positive | 1.00 | 0.50 | 0.30 | 0.00 | | 9.01 | 13,023.7 | 1,886,176 | | 1,710,770 |
|  | 1.00 | 0.50 | 0.40 | 0.00 | | 8.01 | 13,074.3 | 2,242,945 | | 2,029,081 |
|  | 1.00 | 0.50 | 0.50 | 0.00 | | 7.01 | 13,117.0 | 2,591,269 | | 2,344,555 |
|  | 1.00 | 0.50 | 0.60 | 0.00 | | 7.01 | 13,154.0 | 2,933,368 | | 2,657,938 |
|  | 1.00 | 0.50 | 0.70 | 0.00 | | 6.01 | 13,187.0 | 3,270,782 | | 2,969,746 |
|  | 1.00 | 0.50 | 0.80 | 0.00 | | 6.01 | 13,217.1 | 3,604,584 | | 3,280,342 |
|  | 1.00 | 0.50 | 0.90 | 0.00 | | 6.01 | 13,245.0 | 3,935,536 | | 3,589,980 |
|  | 1.00 | 0.50 | 1.00 | 0.00 | | 5.01 | 13,271.4 | 4,264,186 | | 3,898,845 |
|  | 1.00 | 0.60 | 0.30 | 0.00 | | 8.73 | 13,045.9 | 1,946,622 | | 1,774,330 |
|  | 1.00 | 0.60 | 0.40 | 0.00 | | 8.01 | 13,094.4 | 2,302,002 | | 2,092,175 |
|  | 1.00 | 0.60 | 0.50 | 0.00 | | 7.01 | 13,135.4 | 2,649,025 | | 2,407,212 |
|  | 1.00 | 0.60 | 0.60 | 0.00 | | 6.01 | 13,170.9 | 2,989,902 | | 2,720,184 |
|  | 1.00 | 0.60 | 0.70 | 0.00 | | 6.01 | 13,202.6 | 3,326,162 | | 3,031,606 |
|  | 1.00 | 0.60 | 0.80 | 0.00 | | 6.01 | 13,231.5 | 3,658,871 | | 3,341,834 |
|  | 1.00 | 0.60 | 0.90 | 0.00 | | 5.01 | 13,258.2 | 3,988,781 | | 3,651,122 |
|  | 1.00 | 0.60 | 1.00 | 0.00 | | 5.01 | 13,283.5 | 4,316,435 | | 3,959,653 |
|  | 1.00 | 0.70 | 0.20 | 0.00 | | 9.84 | 13,011.5 | 1,641,497 | | 1,516,622 |
|  | 1.00 | 0.70 | 0.30 | 0.00 | | 8.28 | 13,068.8 | 2,006,903 | | 1,837,836 |
|  | 1.00 | 0.70 | 0.40 | 0.00 | | 7.65 | 13,115.3 | 2,360,843 | | 2,155,197 |
|  | 1.00 | 0.70 | 0.50 | 0.00 | | 7.01 | 13,154.4 | 2,706,517 | | 2,469,780 |
|  | 1.00 | 0.70 | 0.60 | 0.00 | | 6.01 | 13,188.4 | 3,046,125 | | 2,782,327 |
|  | 1.00 | 0.70 | 0.70 | 0.00 | | 6.01 | 13,218.7 | 3,381,188 | | 3,093,346 |
|  | 1.00 | 0.70 | 0.80 | 0.00 | | 6.01 | 13,246.3 | 3,712,763 | | 3,403,193 |
|  | 1.00 | 0.70 | 0.90 | 0.00 | | 5.01 | 13,271.9 | 4,041,592 | | 3,712,118 |
|  | 1.00 | 0.70 | 1.00 | 0.00 | | 5.01 | 13,296.1 | 4,368,211 | | 4,020,301 |
|  | 1.00 | 0.80 | 0.20 | 0.00 | | 9.56 | 13,037.9 | 1,703,190 | | 1,580,601 |
|  | 1.00 | 0.80 | 0.30 | 0.00 | | 8.01 | 13,092.5 | 2,067,001 | | 1,901,279 |
|  | 1.00 | 0.80 | 0.40 | 0.00 | | 7.01 | 13,136.9 | 2,419,443 | | 2,218,137 |
|  | 1.00 | 0.80 | 0.50 | 0.00 | | 7.01 | 13,174.2 | 2,763,712 | | 2,532,249 |
|  | 1.00 | 0.80 | 0.60 | 0.00 | | 6.01 | 13,206.6 | 3,101,999 | | 2,844,352 |
|  | 1.00 | 0.80 | 0.70 | 0.00 | | 6.01 | 13,235.5 | 3,435,816 | | 3,154,952 |
|  | 1.00 | 0.80 | 0.80 | 0.00 | | 5.01 | 13,261.8 | 3,766,208 | | 3,464,403 |
|  | 1.00 | 0.80 | 0.90 | 0.00 | | 5.01 | 13,286.1 | 4,093,911 | | 3,772,950 |
|  | 1.00 | 0.80 | 1.00 | 0.00 | | 5.01 | 13,309.1 | 4,419,453 | | 4,080,771 |
|  | 1.00 | 0.90 | 0.20 | 0.00 | | 9.24 | 13,065.4 | 1,764,745 | | 1,644,534 |
|  | 1.00 | 0.90 | 0.30 | 0.00 | | 8.01 | 13,117.3 | 2,126,892 | | 1,964,653 |
|  | 1.00 | 0.90 | 0.40 | 0.00 | | 7.01 | 13,159.4 | 2,477,770 | | 2,280,986 |
|  | 1.00 | 0.90 | 0.50 | 0.00 | | 6.86 | 13,194.8 | 2,820,570 | | 2,594,605 |
|  | 1.00 | 0.90 | 0.60 | 0.00 | | 6.01 | 13,225.6 | 3,157,476 | | 2,906,243 |
|  | 1.00 | 0.90 | 0.70 | 0.00 | | 6.01 | 13,252.9 | 3,489,988 | | 3,216,406 |
|  | 1.00 | 0.90 | 0.80 | 0.00 | | 5.01 | 13,277.8 | 3,819,142 | | 3,525,440 |
|  | 1.00 | 0.90 | 0.90 | 0.00 | | 5.01 | 13,300.9 | 4,145,666 | | 3,833,591 |
| Positive | 1.00 | 0.90 | 1.00 | 0.00 | | 5.01 | 13,322.7 | 4,470,079 | | 4,141,033 |
|  | 1.00 | 1.00 | 0.20 | 0.00 | | 9.01 | 13,094.0 | 1,826,144 | | 1,708,415 |
|  | 1.00 | 1.00 | 0.30 | 0.00 | | 8.01 | 13,143.1 | 2,186,547 | | 2,027,948 |
|  | 1.00 | 1.00 | 0.40 | 0.00 | | 7.01 | 13,182.9 | 2,535,782 | | 2,343,730 |
|  | 1.00 | 1.00 | 0.50 | 0.00 | | 6.01 | 13,216.3 | 2,877,038 | | 2,656,830 |
|  | 1.00 | 1.00 | 0.60 | 0.00 | | 6.01 | 13,245.4 | 3,212,491 | | 2,967,980 |
|  | 1.00 | 1.00 | 0.70 | 0.00 | | 5.01 | 13,271.2 | 3,543,629 | | 3,277,681 |
|  | 1.00 | 1.00 | 0.80 | 0.00 | | 5.01 | 13,294.7 | 3,871,480 | | 3,586,278 |
|  | 1.00 | 1.00 | 0.90 | 0.00 | | 5.01 | 13,316.5 | 4,196,761 | | 3,894,011 |
|  | 1.00 | 1.00 | 1.00 | 0.00 | | 5.01 | 13,337.0 | 4,519,983 | | 4,201,053 |

^*^The parameters were set to the best set of parameter estimates; the frequency of chemotherapy was once a year; each control strategy was simulated for 60 years. $C_{d}$, $C_{e}$, $C_{m}$ indicate the coverage of environmental modification, IEC (focus both on improvement of hygiene habits and changing people’s behavior of raw-fish-consumption) and chemotherapy, respectively.$R_{c}$ is the control reproduction number, $Y_{5\%}$ indicates the years from the beginning of intervention to infection control. DALYs is the disability-adjusted life years. Costs (PZQ) and Costs (ABZ) indicate the total costs of interventions when the drug was praziquantel or albendazole. The underlined texts indicate that the corresponding interventions has costs per DALY averted (compared to that without intervention) less than 1/5 of the willingness-to-pay threshold.
