# Supplementary material for: Cost-effectiveness evaluation of different control strategies for Clonorchis sinensis infection in a high endemic area of China: A modelling study
Source: PLoS Negl Trop Dis. 2022 May 23;16(5):e0010429. doi: 10.1371/journal.pntd.0010429 (PMC9166357; doi:10.1371/journal.pntd.0010429)
Supplement: S8 Table — (DOCX) [file pntd.0010429.s009.docx]

**S8 Table** **Simulation results under the effective control strategies that could reach transmission control within 10 years^*^.**

| Targeted population | Strategy | | |  | Effectiveness | | |  | Total costs/US $ | |
| --- | --- | --- | --- | --- | --- | --- | --- | --- | --- | --- |
|  | $C_{d}$ | $C_{e}$ | $C_{m}$ |  | $R_{c}$ | $Y_{1\%}$ | Averted DALYs |  | Costs (PZQ) | Costs (ABZ) |
| Whole | 0.90 | 1.00 | 1.00 | 0.46 | | 9.01 | 13,316.0 | 6,027,663 | | 2,697,573 |
|  | 1.00 | 0.40 | 1.00 | 0.00 | | 9.01 | 13,238.3 | 5,676,753 | | 2,346,699 |
|  | 1.00 | 0.50 | 0.90 | 0.00 | | 9.01 | 13,232.0 | 5,240,242 | | 2,243,198 |
|  | 1.00 | 0.50 | 1.00 | 0.00 | | 9.01 | 13,257.7 | 5,741,901 | | 2,411,840 |
|  | 1.00 | 0.60 | 0.90 | 0.00 | | 9.01 | 13,251.9 | 5,305,389 | | 2,308,339 |
|  | 1.00 | 0.60 | 1.00 | 0.00 | | 9.01 | 13,276.8 | 5,807,049 | | 2,476,982 |
|  | 1.00 | 0.70 | 0.80 | 0.00 | | 9.01 | 13,246.1 | 4,868,878 | | 2,204,838 |
|  | 1.00 | 0.70 | 0.90 | 0.00 | | 9.01 | 13,271.7 | 5,370,537 | | 2,373,481 |
|  | 1.00 | 0.70 | 1.00 | 0.00 | | 9.01 | 13,295.8 | 5,872,198 | | 2,542,125 |
|  | 1.00 | 0.80 | 0.80 | 0.00 | | 9.01 | 13,266.7 | 4,934,024 | | 2,269,980 |
|  | 1.00 | 0.80 | 0.90 | 0.00 | | 9.01 | 13,291.3 | 5,435,685 | | 2,438,623 |
|  | 1.00 | 0.80 | 1.00 | 0.00 | | 8.01 | 13,314.7 | 5,937,347 | | 2,607,267 |
|  | 1.00 | 0.90 | 0.70 | 0.00 | | 9.01 | 13,262.0 | 4,497,512 | | 2,166,478 |
|  | 1.00 | 0.90 | 0.80 | 0.00 | | 9.01 | 13,287.4 | 4,999,171 | | 2,335,122 |
|  | 1.00 | 0.90 | 0.90 | 0.00 | | 9.01 | 13,311.0 | 5,500,833 | | 2,503,766 |
|  | 1.00 | 0.90 | 1.00 | 0.00 | | 8.01 | 13,333.4 | 6,002,496 | | 2,672,410 |
|  | 1.00 | 1.00 | 0.70 | 0.00 | | 9.01 | 13,284.0 | 4,562,658 | | 2,231,620 |
|  | 1.00 | 1.00 | 0.80 | 0.00 | | 9.01 | 13,308.1 | 5,064,319 | | 2,400,264 |
|  | 1.00 | 1.00 | 0.90 | 0.00 | | 8.01 | 13,330.7 | 5,565,981 | | 2,568,908 |
|  | 1.00 | 1.00 | 1.00 | 0.00 | | 8.01 | 13,352.1 | 6,067,645 | | 2,737,553 |
| Positive | 1.00 | 0.20 | 1.00 | 0.00 | | 9.01 | 13,237.2 | 4,105,068 | | 3,715,626 |
|  | 1.00 | 0.30 | 1.00 | 0.00 | | 9.01 | 13,248.3 | 4,158,465 | | 3,776,820 |
|  | 1.00 | 0.40 | 0.90 | 0.00 | | 9.01 | 13,232.2 | 3,881,902 | | 3,528,708 |
|  | 1.00 | 0.40 | 1.00 | 0.00 | | 9.01 | 13,259.7 | 4,211,516 | | 3,837,896 |
|  | 1.00 | 0.50 | 0.90 | 0.00 | | 9.01 | 13,245.0 | 3,935,536 | | 3,589,980 |
|  | 1.00 | 0.50 | 1.00 | 0.00 | | 9.01 | 13,271.4 | 4,264,186 | | 3,898,845 |
|  | 1.00 | 0.60 | 0.90 | 0.00 | | 9.01 | 13,258.2 | 3,988,781 | | 3,651,122 |
|  | 1.00 | 0.60 | 1.00 | 0.00 | | 9.01 | 13,283.5 | 4,316,435 | | 3,959,653 |
|  | 1.00 | 0.70 | 0.80 | 0.00 | | 9.01 | 13,246.3 | 3,712,763 | | 3,403,193 |
|  | 1.00 | 0.70 | 0.90 | 0.00 | | 9.01 | 13,271.9 | 4,041,592 | | 3,712,118 |
|  | 1.00 | 0.70 | 1.00 | 0.00 | | 9.01 | 13,296.1 | 4,368,211 | | 4,020,301 |
|  | 1.00 | 0.80 | 0.80 | 0.00 | | 9.01 | 13,261.8 | 3,766,208 | | 3,464,403 |
|  | 1.00 | 0.80 | 0.90 | 0.00 | | 9.01 | 13,286.1 | 4,093,911 | | 3,772,950 |
|  | 1.00 | 0.80 | 1.00 | 0.00 | | 9.01 | 13,309.1 | 4,419,453 | | 4,080,771 |
|  | 1.00 | 0.90 | 0.70 | 0.00 | | 9.01 | 13,252.9 | 3,489,988 | | 3,216,406 |
|  | 1.00 | 0.90 | 0.80 | 0.00 | | 9.01 | 13,277.8 | 3,819,142 | | 3,525,440 |
|  | 1.00 | 0.90 | 0.90 | 0.00 | | 9.01 | 13,300.9 | 4,145,666 | | 3,833,591 |
|  | 1.00 | 0.90 | 1.00 | 0.00 | | 8.01 | 13,322.7 | 4,470,079 | | 4,141,033 |
|  | 1.00 | 1.00 | 0.70 | 0.00 | | 9.01 | 13,271.2 | 3,543,629 | | 3,277,681 |
|  | 1.00 | 1.00 | 0.80 | 0.00 | | 9.01 | 13,294.7 | 3,871,480 | | 3,586,278 |
|  | 1.00 | 1.00 | 0.90 | 0.00 | | 8.01 | 13,316.5 | 4,196,761 | | 3,894,011 |
|  | 1.00 | 1.00 | 1.00 | 0.00 | | 8.01 | 13,337.0 | 4,519,983 | | 4,201,053 |

^*^The parameters were set to the best set of parameter estimates; the frequency of chemotherapy was once a year; each control strategy was simulated for 60 years. $C_{d}$, $C_{e}$, $C_{m}$ indicate the coverage of environmental modification, IEC (focus both on improvement of hygiene habits and changing people’s behavior of raw-fish-consumption) and chemotherapy, respectively.$R_{c}$ is the control reproduction number, $Y_{1\%}$ indicates the years from the beginning of intervention to transmission control. DALYs is the disability-adjusted life years. Costs (PZQ) and Costs (ABZ) indicate the total costs of interventions when the drug was praziquantel or albendazole. The underlined texts indicate that the corresponding interventions has costs per DALY averted (compared to that without intervention) less than 1/5 of the willingness-to-pay threshold.
