# Supplementary material for: Cost-effectiveness evaluation of different control strategies for Clonorchis sinensis infection in a high endemic area of China: A modelling study
Source: PLoS Negl Trop Dis. 2022 May 23;16(5):e0010429. doi: 10.1371/journal.pntd.0010429 (PMC9166357; doi:10.1371/journal.pntd.0010429)
Supplement: S9 Table — (DOCX) [file pntd.0010429.s010.docx]

**S9 Table** **The optimal cost-effective strategies with targeted population of any types and the upper coverage set to 90%^*^.**

| Control type | Drug | The optimal strategy | | | | |
| --- | --- | --- | --- | --- | --- | --- |
|  |  | Applied population of chemotherapy | $C_{d}$ | $C_{e}$ | $C_{m}$ | Proportion (%) |
| Infection control | PZQ | Positive | 0.90 | 0.90 | 0.90 | 400 (80.0) |
|  |  | At-risk | 0.90 | 0.90 | 0.90 | 87 (17.4) |
|  | ABZ | Whole | 0.90 | 0.90 | 0.90 | 466 (93.2) |
| Transmission control | PZQ | Positive | 0.90 | 0.90 | 0.90 | 258 (51.6) |
|  |  | Whole | 0.90 | 0.90 | 0.90 | 174 (34.8) |
|  | ABZ | Whole | 0.90 | 0.90 | 0.90 | 326 (65.2) |

^*^Only strategies with proportion among 500 simulations ≥10% were displayed. The frequency of chemotherapy was once a year, and the intervention duration was 10 years.
