# Supplementary material for: Cost-effectiveness evaluation of different control strategies for Clonorchis sinensis infection in a high endemic area of China: A modelling study
Source: PLoS Negl Trop Dis. 2022 May 23;16(5):e0010429. doi: 10.1371/journal.pntd.0010429 (PMC9166357; doi:10.1371/journal.pntd.0010429)
Supplement: S10 Table — (DOCX) [file pntd.0010429.s011.docx]

**S10 Table The optimal cost-effective strategies to reach infection control for other targeted population of chemotherapy^*^**

| Drug | Targeted population of chemotherapy | The optimal strategy | | | |
| --- | --- | --- | --- | --- | --- |
|  |  | $C_{d}$ | $C_{e}$ | $C_{m}$ | Proportion (%) |
| PZQ | Whole | 1.00 | 1.00 | 0.50 | 153 (30.6) |
|  |  | 1.00 | 1.00 | 0.40 | 114 (22.8) |
|  |  | 1.00 | 1.00 | 0.60 | 111 (22.2) |
|  | At-risk | 1.00 | 1.00 | 1.00 | 421 (84.2) |
|  |  | 1.00 | 1.00 | 0.90 | 53 (10.6) |
|  | Positive | 1.00 | 1.00 | 1.00 | 175 (35.0) |
|  |  | 1.00 | 1.00 | 0.80 | 92 (18.4) |
|  |  | 1.00 | 1.00 | 0.70 | 78 (15.6) |
|  |  | 1.00 | 1.00 | 0.60 | 69 (13.8) |
|  |  | 1.00 | 1.00 | 0.90 | 50 (10.0) |
| ABZ | Whole | 1.00 | 1.00 | 1.00 | 499 (99.8) |
|  | At-risk | 1.00 | 1.00 | 1.00 | 444 (88.8) |
|  | Positive | 1.00 | 1.00 | 1.00 | 207 (41.4) |
|  |  | 1.00 | 1.00 | 0.80 | 79 (15.8) |
|  |  | 1.00 | 1.00 | 0.70 | 77 (15.4) |
|  |  | 1.00 | 1.00 | 0.90 | 69 (13.8) |

^*^Only strategies with proportions≥10% were presented. The frequency of chemotherapy was once a year, and the intervention duration was 10 years.
