# Supplementary material for: Cost-effectiveness evaluation of different control strategies for Clonorchis sinensis infection in a high endemic area of China: A modelling study
Source: PLoS Negl Trop Dis. 2022 May 23;16(5):e0010429. doi: 10.1371/journal.pntd.0010429 (PMC9166357; doi:10.1371/journal.pntd.0010429)
Supplement: S11 Table — (DOCX) [file pntd.0010429.s012.docx]

**S11 Table The optimal cost-effective strategies to reach transmission control for other targeted population of chemotherapy^*^**

| Drug | Targeted population of chemotherapy | The optimal strategy | | | |
| --- | --- | --- | --- | --- | --- |
|  |  | $C_{d}$ | $C_{e}$ | $C_{m}$ | Proportion (%) |
| PZQ | Whole | 1.00 | 1.00 | 0.60 | 207 (41.4) |
|  |  | 1.00 | 1.00 | 0.50 | 113 (22.6) |
|  |  | 1.00 | 1.00 | 0.70 | 83 (16.6) |
|  | Positive | 1.00 | 1.00 | 1.00 | 158 (31.6) |
|  |  | 1.00 | 1.00 | 0.80 | 95 (19.0) |
|  |  | 1.00 | 1.00 | 0.70 | 93 (18.6) |
|  |  | 1.00 | 1.00 | 0.60 | 70 (14.0) |
|  |  | 1.00 | 1.00 | 0.90 | 51 (10.2) |
| ABZ | Whole | 1.00 | 1.00 | 1.00 | 486 (97.2) |
|  | Positive | 1.00 | 1.00 | 1.00 | 187 (37.4) |
|  |  | 1.00 | 1.00 | 0.70 | 86 (17.2) |
|  |  | 1.00 | 1.00 | 0.80 | 83 (16.6) |
|  |  | 1.00 | 1.00 | 0.90 | 68 (13.6) |

^*^Only strategies with proportions≥10% were presented. The frequency of chemotherapy was once a year, and the intervention duration was 10 years.
