# Supplementary material for: Cost-effectiveness evaluation of different control strategies for Clonorchis sinensis infection in a high endemic area of China: A modelling study
Source: PLoS Negl Trop Dis. 2022 May 23;16(5):e0010429. doi: 10.1371/journal.pntd.0010429 (PMC9166357; doi:10.1371/journal.pntd.0010429)
Supplement: S1 File — (DOCX) [file pntd.0010429.s013.docx]

# S1 File. Transmission model

We refined the basic model to describe the transmission of *C. sinensis* based on our previous work [1]. In order to better integrate with the cost-effectiveness analysis, the parameter of disease fatality rate was newly incorporated into the previous multi-group dynamic transmission model. By using the survey data of Fusha Town, Guangdong Province, we estimated the unknown model parameters based on Bayesian melding approach. By adding specific intervention parameters to the basic model, a full model with interventions was further developed.

## A. The basic model

We refined the basic model based on our previous work to describe the transmission of *C. sinensis* among snails, fish and different groups of people with different raw-fish-consumption behaviors [1]. We assumed $S_{h,g}$, $I_{h,g}$, $S_{s}$, $I_{s}$, $S_{f}$ and $I_{f}$ represent the numbers of susceptible humans, infected humans, susceptible snails, infected snails, susceptible fish and infected fish, respectively, where $g=1, 2, 3, 4$ respectively represent the human groups that eats raw fish seldom, moderately (<5 times per year), often (5-10 times per year), and very often (>10 times per year). The ordinary differential equations describing the model are shown as follows:

$$\left\{ \begin{aligned} &\frac{dS_{h,g}}{dt}=\lambda_{h,g}-c_{g}\beta_{h,1}S_{h,g}I_{f}-\mu_{h}S_{h,g}+\gamma_{1}I_{h,g}, \\ &\frac{dI_{h,g}}{dt}={c_{g}\beta}_{h,1}S_{h,g}I_{f}-\mu_{h}I_{h,g}-\mu_{d}I_{h,g}-\gamma_{1}I_{h,g}, \\ &\frac{dS_{s}}{dt}=\lambda_{s}-\beta_{s}S_{s}\left( I_{h,1}+I_{h,2}+I_{h,3}+I_{h,4} \right)-\mu_{s}S_{s}, \\ &\frac{dI_{s}}{dt}=\beta_{s}S_{s}\left( I_{h,1}+I_{h,2}+I_{h,3}+I_{h,4} \right)-\mu_{s}I_{s}, \\ &\frac{dS_{f}}{dt}=\lambda_{f}-\beta_{f}S_{f}I_{s}-\mu_{f}S_{f}, \\ &\frac{dI_{f}}{dt}=\beta_{f}S_{f}I_{s}-\mu_{f}I_{f}, \end{aligned} \right.$$

Descriptions of model parameters are listed in **S1 Table**. The numbers of humans, snails and fish transferring from susceptible to infected state are $c_{g}\beta_{h,1}S_{h,g}I_{f}$ $(g=1, 2, 3, 4$ and $c_{1}=1$), $\beta_{s}S_{s}\left( I_{h,1}+I_{h,2}+I_{h,3}+I_{h,4} \right)$ and $\beta_{f}S_{f}I_{s}$, respectively, while the number of humans transferring back after recovery is $\gamma_{1}I_{h,g}(g=1,2,3,4)$. Hosts enter into the system as susceptible individuals with recruitment number $\lambda$, and leave it with death rate $\mu$. To better integrate with the cost-effectiveness analysis, we made a refinement to the previous model by incorporating death rate from *C. sinensis* infections to the overall death for infected humans. The death rate for infected humans in the current model includes natural death rate $\mu_{h}$ and death rate from *C. sinensis* infections $\mu_{d}$.

**Supplementary Table 1.** **Descriptions of model parameters (unit: day^-1^).**

| Parameter | Description |
| --- | --- |
| $\lambda_{h,1}$ | Recruitment of susceptible humans who seldom eat raw or uncooked fish |
| $\lambda_{h,2}$ | Recruitment of susceptible humans who moderately eat raw or uncooked fish |
| $\lambda_{h,3}$ | Recruitment of susceptible humans who often eat raw or uncooked fish |
| $\lambda_{h,4}$ | Recruitment of susceptible humans who very often eat raw or uncooked fish |
| $\lambda_{s}$ | Recruitment of susceptible snails |
| $\lambda_{f}$ | Recruitment of susceptible fish |
| $\beta_{h,1}$ | Transmission rate from an infected fish to a susceptible human who seldom consumes raw fish |
| $c_{2}$ | Ratio of transmission rate from an infected fish to a susceptible human who moderately consumes raw or uncooked fish to that who seldom |
| $c_{3}$ | Ratio of transmission rate from an infected fish to a susceptible human who often consumes raw or uncooked fish to that who seldom |
| $c_{4}$ | Ratio of transmission rate from an infected fish to a susceptible human who very often consumes raw or uncooked fish to that who seldom |
| $\beta_{h,2}$ | The increased transmission rate from an infected fish to a susceptible human who moderately consumes raw fish, compared to that who seldom, which is equal to ${\beta_{h,1}(c}_{2}-1)$ |
| $\beta_{h,3}$ | The increased transmission rate from an infected fish to a susceptible human who often consumes raw fish, compared to that who seldom, which is equal to ${\beta_{h,1}(c}_{3}-1)$ |
| $\beta_{h,4}$ | The increased transmission rate from an infected fish to a susceptible human who very often consumes raw fish, compared to that who seldom, which is equal to ${\beta_{h,1}(c}_{4}-1)$ |
| $\beta_{s}$ | Transmission rate from an infected human to a susceptible snail |
| $\beta_{f}$ | Transmission rate from an infected snail to a susceptible fish |
| $\mu_{h}$ | Nature birth and death rate of human hosts |
| $\mu_{d}$ | Fatality rate from *C.sinensis* infections |
| $\mu_{s}$ | Birth and death rates of snails |
| $\mu_{f}$ | Birth and death rates of fish |
| $\gamma_{1}$ | Basic recovery rate of infected humans through individual treatment |

## B. Data

As a practical example, we applied the model in Fusha Town, a typical high endemic area in Zhongshan City, Guangdong Province. The source of prevalence data of each host is the same as our previous work [1]. The prevalence data of human is shown in **S2 Table** [2], while the prevalence of fish is 20.97% (39/186) [3]. Since no survey data of snails is available for either Fusha Town or Zhongshan City, the range of prevalence of infected snails in Guangdong Province (0.1% to 3.7%) was set as prior information for the following parameter estimation [3,4].

**Supplementary Table 2.** **Observed prevalence of *C. sinensis* infection among groups of people with different frequencies of raw fish consumption in Fusha Town.**[2]

| Frequency of eating raw fish per year | No. of samples | No. of positive (%) |
| --- | --- | --- |
| Seldom | 802 | 121 (15.08) |
| <5 times | 215 | 126 (58.60) |
| 5-10 times | 123 | 98 (79.67) |
| >10 times | 60 | 59 (98.33) |
| Total | 1200 | 404 (33.67) |

## C. Parameter estimation

We adopted Bayesian melding approach to estimate the unknown parameters based on the basic model and the survey data. Triangular distributions were used to set prior information obtained from different sources for unknown parameters, and the modes and ranges of prior and posterior distributions are shown in **S3 Table**. We assumed the numbers of entering (*λ*) and leaving the system (*μ*) were the same, thus $\lambda_{h,g}=\mu_{h}N_{h,g}+\mu_{d}I_{h,g}(g=1,2,3,4)$. The fatality rate from *C. sinensis* infections $\mu_{d}$ was set equal to the incidence rate of cholangiocarcinoma attributable to *C. sinensis* infections, due to the close relationship between death of patients with clonorchiasis and cholangiocarcinoma and the poor prognosis of patients with cholangiocarcinoma [5]. Therefore, $\mu_{d}=I_{0}*(OR-1)$, where $I_{0}$ is the incidence rate of cholangiocarcinoma in non-endemic area of *C. sinensis* infection, and $OR$ is the odds ratio between *C. sinensis* infection and cholangiocarcinoma [5,6,7]. The best set of estimated parameters was recognized as the one with the largest posterior density and 500 posterior sets of estimated parameters of the basic model were obtained by Sampling Importance Resampling (SIR) method [8]. Other details of parameter estimation and model validation have been explained in our previously published paper [1].

**Supplementary Table 3.** **The prior distributions and posterior estimations of model parameters (unit: day^-1^).**

| Parameter | Prior | |  | Posterior | |
| --- | --- | --- | --- | --- | --- |
|  | Mode [Range] | Source |  | Best set [95% CI] | Source |
| $p_{1}$ | 0.67 [0.57-0.77] | [2] |  | 0.67 [0.65-0.69] | fitting |
| $p_{2}$ | 0.18 [0.08-0.28] | [2] |  | 0.18 [0.16-0.20] | fitting |
| $p_{3}$ | 0.103 [0.053-0.153] | [2] |  | 0.100 [0.088-0.118] | fitting |
| $p_{4}$ | 0.050 [0.040-0.060] | [2] |  | 0.050 [0.044-0.055] | fitting |
| $\lambda_{h1}$ | $p_{1}^{'}\times N_{h}\times\mu_{h}+{\mu_{d}\times I}_{h1}$* | |  | 0.58 [0.56-0.60] | fitting |
| $\lambda_{h2}$ | $p_{2}^{'}\times N_{h}\times\mu_{h}+{\mu_{d}\times I}_{h2}$ | |  | 0.15 [0.14-0.17] | fitting |
| $\lambda_{h3}$ | $p_{3}^{'}\times N_{h}\times\mu_{h}+{\mu_{d}\times I}_{h3}$ | |  | 0.087 [0.077-0.103] | fitting |
| $\lambda_{h4}$ | $p_{4}^{'}\times N_{h}\times\mu_{h}+{\mu_{d}\times I}_{h4}$ | |  | 0.044 [0.039-0.049] | fitting |
| $\lambda_{s}$ | - | - |  | 3729.1 | [9,10,11] |
| $\lambda_{f}$ | 2191.78 [219.18-4383.56] | [10,12,13] |  | 2598.6 [1277.7-3272.8] | fitting |
| $\beta_{h1}$ | 2.81×10^-10^ [2.81×10^-11^-5.63×10^-10^] | Solving equations |  | 3.01×10^-10^ [1.40×10^-10^-4.06×10^-10^] | fitting |
| $\beta_{s}$ | 2.71×10^-9^ [2.71×10^-10^-5.43×10^-9^] | Same as $\beta_{h1}$ |  | 2.07×10^-9^ [1.64×10^-9^-3.95×10^-9^] | fitting |
| $\beta_{f}$ | 1.87×10^-8^ [1.87×10^-9^-3.75×10^-8^] | Same as $\beta_{h1}$ |  | 2.13×10^-8^ [1.14×10^-8^-2.82×10^-8^] | fitting |
| $c_{2}$ | 8 [3-13] | $\frac{{I_{h,2}}/{S_{h,2}}}{{I_{h,1}}/{S_{h,1}}}$ |  | 8.13 [6.23-10.51] | fitting |
| $c_{3}$ | 22 [17-32] | $\frac{{I_{h,3}}/{S_{h,3}}}{{I_{h,1}}/{S_{h,1}}}$ |  | 23.29 [18.63-28.30] | fitting |
| $c_{4}$ | 332 [312-352] | $\frac{{I_{h,4}}/{S_{h,4}}}{{I_{h,1}}/{S_{h,1}}}$ |  | 332.30 [320.52-346.53] | fitting |
| $\mu_{h}$ | - | - |  | 1.49×10^-5^ | [14] |
| $\mu_{d}$ | 2.10×10-7 [1.38×10-7-3.12×10-7] | [5,6,7] |  | 2.18×10^-7^ [1.65×10^-7^-2.86×10^-7^] | fitting |
| $\mu_{s}$ | - | - |  | 1/365 | [11] |
| $\mu_{f}$ | 1/(1.5×365) [1/(3×365)- 1/(1×365)] | [12] |  | 1/(1.62×365) [1/(2.06×365)-1/(1.15×365)] | fitting |
| $\gamma_{1}$ | 0.140/365 [0.014/365-0.200/365] | [15] |  | 0.172/365 [0.068/365-0.173/365)] | fitting |

*$p_{g}^{'} (g=1,2,3,4)$ represents adjusted proportion of $p_{g}$, to make the sum of $p_{g}^{'}$ for all groups equals to one.

# References

1. Huang X, Qian M, Zhu G, Fang Y, Hao Y, Lai Y. Assessment of control strategies against Clonorchis sinensis infection based on a multi-group dynamic transmission model. Plos Neglect Trop D. 2020;14(3):e8152. http://doi.org/10.1371/journal.pntd.0008152

2. Du S, Huang J, Li H. [Epidemiology of clonorchiasis in the towns near the Pearl River Delta] (author’s tranl). South China J Prev Med. 2015;41(3):273–5. Chinese.

3. Zhang X, Pei F, Zhang Q, Lin R, Huang S, Wang J, et al. [Current status of environmental sanitation and clonorchis sinensis intermediate host infection of freshwater aquaculture in partial areas of Guangdong Province]. South China J Prev Med. 2010;36(3):9-13. Chinese.

4. Li F, Lin R, Huang B, Zhou Y, Ou B, Luo C, et al. [Epidemiological investigation on clonorchiasis and exploration of treatment measures in urban district of jiangmen city]. Journal of Pathogen Biology 2005;18(3):214-6. Chinese.

5. Fürst T, Keiser J, Utzinger J. Global burden of human food-borne trematodiasis: a systematic review and meta-analysis. Lancet Infect Dis. 2012;12(3):210-21. https://doi.org/10.1016/S1473-3099(11)70294-8

6. Parkin DM. The global health burden of infection-associated cancers in the year 2002. Int J Cancer. 2006;118(12):3030-44. https://doi.org/10.1002/ijc.21731

7. Khan AS, Dageforde LA. Cholangiocarcinoma. Surg Clin North Am. 2019;99(2):315-35. https://doi.org/10.1016/j.suc.2018.12.004

8. Rubin DB. Using the SIR algorithm to simulate posterior distributions. Bayesian Statistics. 1988:395-402.

9. Wang J, Zhou J, Lyu W, Zhang H, Li S, Zheng X, et al. [Effect of aquatic plant coverage and water depth on the diversity of large aquatic animals: A study based on ponds in the lower reaches of the Yangtze River]. Acta Agriculturae Shanghai. 2017;33(3):65-70. Chinese.

10. Xie X [Internet]. [The rent of fish pond in Zhongshan, Guangdong Province is up to 4750 yuan per mu, which breaking the record] (author's tranl). [cited 2021 Oct 27]. Available from: http://www.bbwfish.com/article.asp?artid=86551. Chinese.

11. Chen Z, Zou L, Shen D, Zhang W, Ruan S. Mathematical modelling and control of Schistosomiasis in Hubei Province, China. Acta Trop. 2010;115(1-2):119-25. https://doi.org/10.1016/j.actatropica.2010.02.012

12. Liu J, Zhang J, Yuan H. [The investigation and analysis about cost and benefit of freshwater fish breeding]. Chinese Fisheries Economics. 2017;35(1):18-27. Chinese.

13. Baiduzhidao [Internet]. [How many fish can be raised in one mu of fishpond?] (author's tranl). [cited 2021 Oct 27]. Available from: https://zhidao.baidu.com/question/1430988985257871219.html. Chinese.

14. Zhongshan Statistical Bureau [Internet]. [Zhongshan Statistical Yearbook 2013]. [cited 2021 Oct 27]. Available from: http://stats.zs.gov.cn/tjzl/tjnj/2013nj/index.htm. Chinese.

15. Ma J, Chen G, Tang X, Zuo Z, Cao F, Gong P, et al. [Survey of life habits and health knowledge of the people in epidemic region of Clonorchiasis]. J Trop Med. 2008;8(8):858-60, 865. Chinese.
