# Supplementary material for: Cost-effectiveness evaluation of different control strategies for Clonorchis sinensis infection in a high endemic area of China: A modelling study
Source: PLoS Negl Trop Dis. 2022 May 23;16(5):e0010429. doi: 10.1371/journal.pntd.0010429 (PMC9166357; doi:10.1371/journal.pntd.0010429)
Supplement: S2 File — (DOCX) [file pntd.0010429.s014.docx]

# S2 File. Full model with interventions

With considerations of the chemotherapy compliance and efficacy of interventions, the full model with interventions was modified based on the basic model as follows:


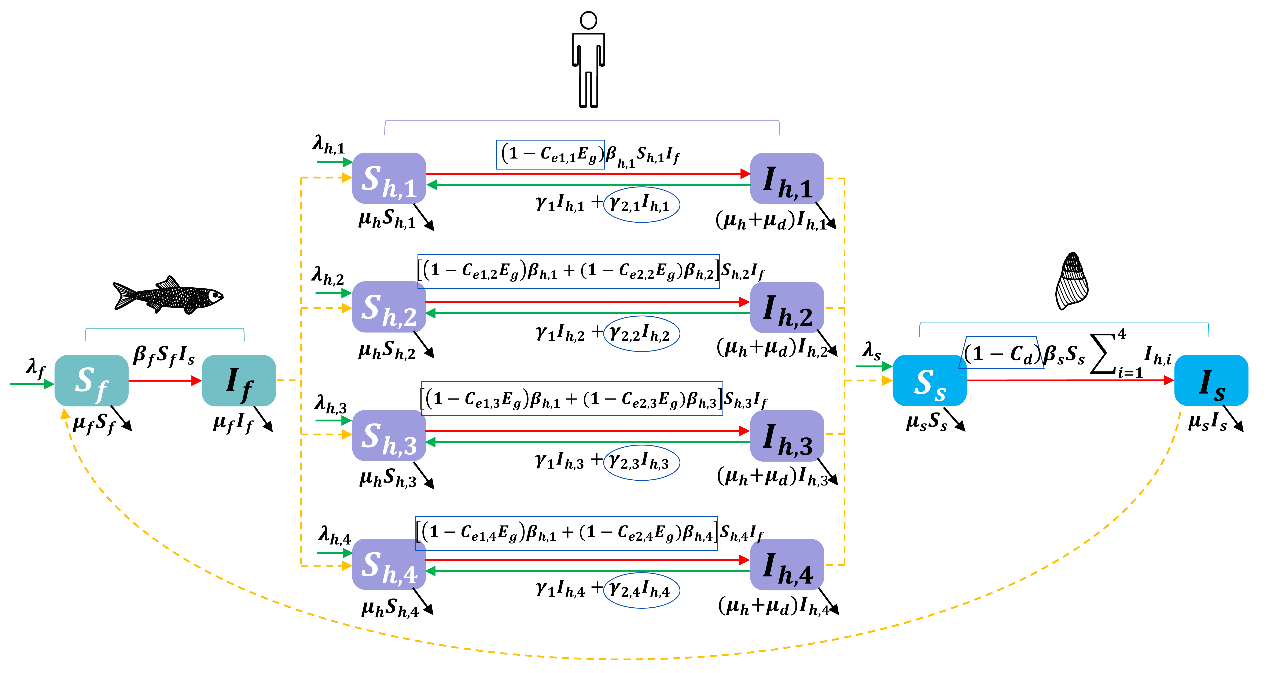


**Supplementary Figure 1.** **The full model of *C. sinensis* infection with interventions**

$$\left\{ \begin{aligned} & \frac{dS_{h,g}}{dt}=\lambda_{h,g}-\left[ \left( 1-C_{e1,g}E_{g} \right)\beta_{h,1}+(1-C_{e2,g}E_{g})\beta_{h,g} \right]S_{h,g}I_{f}-\mu_{h}S_{h,g}+\gamma_{1}I_{h,g}+\gamma_{2,g}I_{h,g}, \\ & \frac{dI_{h,g}}{dt}=\left[ \left( 1-C_{e1,g}E_{g} \right)\beta_{h,1}+(1-C_{e2,g}E_{g})\beta_{h,g} \right]S_{h,g}I_{f}-\mu_{h}I_{h,g}-\mu_{d}I_{h,g}-\gamma_{1}I_{h,g}-\gamma_{2,g}I_{h,g}, \\ & \frac{dS_{s}}{dt}=\lambda_{s}-{(1-C_{d})\beta}_{s}S_{s}\left( I_{h,1}+I_{h,2}+I_{h,3}+I_{h,4} \right)-\mu_{s}S_{s}, \\ & \frac{dI_{s}}{dt}={(1-C_{d})\beta}_{s}S_{s}\left( I_{h,1}+I_{h,2}+I_{h,3}+I_{h,4} \right)-\mu_{s}I_{s}, \\ & \frac{dS_{f}}{dt}=\lambda_{f}-\beta_{f}S_{f}I_{s}-\mu_{f}S_{f}, \\ & \frac{dI_{f}}{dt}=\beta_{f}S_{f}I_{s}-\mu_{f}I_{f}, \end{aligned} \right.$$

Compared to the full model in the previous work, the compliance of chemotherapy ($A_{g}$) was included, which made the recovery rate of infected humans through mass chemotherapy per unit of time described as $\gamma_{2,g}=-\frac{\ln\left( 1-C_{m,g}A_{g}h \right)}{T_{g}}$. $C_{m,g}$ represents the coverage of chemotherapy, namely the proportion of people receiving drugs among the targeted population. $A_{g}$ represents the compliance of chemotherapy, which is the proportion of people actually taking drugs among the population receiving drugs. Therefore, $C_{m,g}A_{g}$ indicates the actual proportion of people receiving treatment among the targeted population. With consideration that the compliance of chemotherapy ($A_{g}$) may be influenced by targeted population (i.e., whole, at-risk or positive) and whether chemotherapy combined with IEC or not [1,2,3], we set the corresponding values of compliance shown in **S4 Table** in the full model. As presented in the previous study, $h$ represents drug efficacy and $T_{g}$represents the time interval of treatment in days. We assumed the drug being praziquantel (75 mg/kg, PZQ) or albendazole (total dose 3.2 g, twice per day for 4 days, ABZ), which are commonly used in China [4]. Drug efficacy was set to be the average efficacy of the two drugs, namely 92%, as there was no significant difference between the two drugs in efficacy [5]. If chemotherapy was targeted on positive population, diagnostic sensitivity should be considered when calculating the recovery rate, namely $\gamma_{2,g}=-\frac{\ln\left( 1-C_{m,g}\times S\times A_{g}\times h \right)}{T_{g}}$, where $S$ represents the sensitivity of the diagnostic approach. We assumed the diagnostic approach being the commonly used Kato-Katz thick smear method with 2 smears for 1 stool [6], and its sensitivity followed a triangular distribution with its mode being 63.6% and the upper and lower bounds being plus or minus 25% of the mode [7]. For IEC, the efficacy of IEC ($E_{g}$) was considered, which indicates the proportion of people improving healthy behaviors among the population receiving IEC. $C_{e1,g}$ and $C_{e2,g}$ indicate the proportions of people who have received information on improving hygiene habits and changing eating habits, respectively, so that $C_{e1,g}E_{g}$ and $C_{e2,g}E_{g}$ indicate the actual improvement rate of hygiene habits and rate of stopping raw-fish-eating behavior, respectively. Since it is not easy to change people’s hygiene and eating habits and these changes may differ in different endemic areas [8], we assumed that the improvement rate of hygiene habit or stopping raw-fish-eating behavior, namely efficacy of IEC (E), being 54.08% under a 100% coverage of IEC [2] and following a triangular distribution with mode being 54.08% and the upper and lower bounds are the mode plus or minus 25% of it. For environmental modification, $C_{d}$ represents the coverage of sanitation toilets.

**Supplementary Table 4.** **Values set for compliance of chemotherapy among different kinds of targeted population.**

| Strategy | | Targeted population | Base value | Range | Distribution | Effective coverage of treatment | Reference |
| --- | --- | --- | --- | --- | --- | --- | --- |
| Chemotherapy | | Whole (${A_{w1}}^{*}$) | 54.76% | Base±25% | Triangular | ${C_{m}}^{\dagger}{A_{w1}}^{*}$ | [1,2,3] |
|  | | At-risk (${A_{k1}}^{*}$) | 71.64% | Base±25% | Triangular | ${C_{m}}^{\dagger}{A_{k1}}^{*}$ | [1] |
|  | | Positive | 100% | - | - | ${C_{m}}^{\dagger}$ | [1] |
| The increased compliance of chemotherapy due to IEC$(A_{incre}$) | ${A_{incre}}^{*}$ | | 31.52% | Base±25% | Triangular | - | [3] |
| Chemotherapy + IEC | | Whole (${A_{w2}}^{*}$) | ${A_{w1}}^{*}(1+C_{e}{A_{incre}}^{*})$ | - | - | ${C_{m}}^{\dagger}{A_{w1}}^{*}(1+{C_{e}}^{\ddagger}{A_{incre}}^{*})$ | - |
|  | | At-risk (${A_{k2}}^{*}$) | ${A_{k1}}^{*}(1+C_{e}{A_{incre}}^{*})$ | - | - | ${C_{m}}^{\dagger}{A_{k1}}^{*}(1+{C_{e}}^{\ddagger}{A_{incre}}^{*})$ | - |
|  | | Positive | 100% | - | - | ${C_{m}}^{\dagger}$ | [1] |

^*^*A* indicates the compliance of chemotherapy.

^†^$C_{m}$ is the coverage of chemotherapy.

^‡^$C_{e}$ is the coverage of IEC.

The calculation formula of control reproduction number ($R_{c}$) is similar as the previous study, which is expressed as following:

$$R_{c}=\sqrt[3]{\frac{\lambda_{s}{{\lambda_{f}\beta}_{s}\beta}_{f}(1-C_{d})}{\mu_{s}^{2}\mu_{f}^{2}}\sum_{g=1}^{4} \frac{N_{h,g}\left[ (1-C_{e1,g})\beta_{h,1}+(1-C_{e2,g})\beta_{h,g} \right]}{\mu_{h}+\mu_{d}+\gamma_{1}+\gamma_{2,g}}}$$

We obtained the best set of estimated parameters of full model by combining the mode of the intervention parameters with the best parameter set of the basic model. 500 estimated parameter vectors of full model were obtained by combining 500 parameter sets randomly sampled from distribution of intervention parameters with 500 posterior parameter sets of the basic model.

# References

1. Fang Y, Ruan C, Gao X, Tan Q, Chen R, Hao Y. [Research on chemotherapy measures in different Clonorchis sinensis infectiosity endemic areas]. Chin J Schisto Control. 2014;26(3):300-2. Chinese.

2. Tan Q, Yu Z, Ma J, Tang X, Gong F, Cao F, et al. [Evaluation of health education and chemotherapeutic treatment on the prevention of clonorchiasis]. J Trop Med. 2012;12(4):478-80. Chinese.

3. Hu G, Hu J, Song K, Lin D, Zhang J, Cao C, et al. The role of health education and health promotion in the control of schistosomiasis: experiences from a 12-year intervention study in the Poyang Lake area. Acta Trop. 2005;96(2-3):232-41. https://doi.org/10.1016/j.actatropica.2005.07.016

4. Huang X, Fang Y, Lai Y. [A review on population control strategies of clonorchiasis in China]. J Trop Med. 2020;20(2):267-74. Chinese.

5. Feng Q, Luo L, Xia S, Zhang R, Wu H, Fan M, et al. [Compare the efficacy of two antiparasitic drugs against Clonorchiasis]. J Trop Med. 2006;6(12):1291-2. Chinese.

6. Qian M, Zhou C, Zhu H, Chen Y, Zhou X. Comparison on different treatment strategies against Clonorchis sinensis infection. Infect Dis Poverty. Forthcoming 2021.

7. Qian M, Zhuang S, Zhu S, Deng X, Li Z, Zhou X. Improving diagnostic performance of the Kato‑Katz method for Clonorchis sinensis infection through multiple samples. Parasites & Vectors. 2019;12(1): 336. https://doi.org/10.1186/s13071-019-3594-5

8. Yu Y, Wu K. [Retrospective analysis of epidemiological investigations of clonorchiasis conducted within the past 20 years in Shunde of Guangdong]. Modern Preventive Medicine. 2015;42(22):4050-2, 4080.
