# Supplementary material for: Cost-effectiveness evaluation of different control strategies for Clonorchis sinensis infection in a high endemic area of China: A modelling study
Source: PLoS Negl Trop Dis. 2022 May 23;16(5):e0010429. doi: 10.1371/journal.pntd.0010429 (PMC9166357; doi:10.1371/journal.pntd.0010429)
Supplement: S3 File — (DOCX) [file pntd.0010429.s015.docx]

# **S3 File. Three recommended strategies**

WHO recommended preventive chemotherapy with praziquantel (40 mg/kg) for all residents every year in high endemic areas (with prevalence≥20%). In moderate endemic areas (with prevalence <20%), it is recommended to perform chemotherapy for all residents every two years or for individuals regularly eating raw fish every year [1]. The Chinese government recommended a combination of control strategies composing of chemotherapy with 80% coverage for at-risk population every year and improvement of sanitation toilets with 80% coverage [2]. The government of Guangdong Province, which is one of the most endemic provinces in China, recommended stronger interventions, including chemotherapy with 80% coverage targeted on at-risk population every year, sanitation toilets with 90% coverage and improvement of people’s healthy behaviors with 90% coverage [3]. Values of intervention parameters of three recommended strategies are shown in **S5 Table**.

**Supplementary Table 5.** **Values of intervention parameters of the current recommended control strategies^*^.**

| Parameter^#^ | WHO | The Chinese government | The government of Guangdong Province |
| --- | --- | --- | --- |
| $C_{d}$ | 0 | 0.85 | 0.90 |
| $C_{e1,g}(g=1,2,3,4)$ | 0 | 0 | 0.90 |
| $C_{e2,g}(g=2,3,4)$ | 0 | 0 | 0.90 |
| $C_{m,1}$ | 1.00 | 0 | 0 |
| $C_{m,2}$ | 1.00 | 0.80 | 0.80 |
| $C_{m,3}$ | 1.00 | 0.80 | 0.80 |
| $C_{m,4}$ | 1.00 | 0.80 | 0.80 |
| *F* | 1 | 1 | 1 |
| *D* | 10 | 10 | 10 |

^*^The Chinese government do have a strategy through IEC to improve the awareness of key knowledge on clonorchiasis control among students by 95%. However, such improvement does not have a direct effect on the system and is difficult to address. Thus, we set $C_{e1,g}=0 (g=1,2,3,4)$ and $C_{e2,g}=0 (g=2,3,4)$.

^#^$C_{d}$ is the coverage of sanitation toilets, $C_{e1,g}$ is the proportions of people who have received information on improving hygiene habits, $C_{e2,g}$ is the proportions of people who have received information on stopping raw-fish-eating behavior and $C_{m, g}$ is the coverage of chemotherapy. $g=1,2,3,4$ represent human groups who seldom, moderately, often and very often consume raw fish, respectively. *F* indicates the frequency of chemotherapy per year and *D* indicates the intervention duration of chemotherapy.

# References

1. World Health Organization [Internet]. Sustaining the drive to overcome the global impact of neglected tropical diseases: second WHO report on neglected tropcal diseases; c2019 [cited 2021 Oct 27]. Available from: https://www.who.int/neglected_diseases/9789241564540/en/.

2. National Health and Family Planning Commission of the People’s Republic of China [Internet]. National control plan on echinococcosis and other important parasitic diseases in China (2016–2020). [cited 2021 Oct 27]. Available from: http://www.nhc.gov.cn/jkj/s5873/201702/dda5ffe3f50941a29fb0aba6233bb497.shtml. Chinese.

3. Health and Family Planning Commission of Guangdong Province [Internet]. Control plan on the important parasitic diseases in Guangdong Province, China (2016-2020). [cited 2021 Oct 27]. Available from:http://zwgk.gd.gov.cn/0069401
