# Supplementary material for: Cost-effectiveness evaluation of different control strategies for Clonorchis sinensis infection in a high endemic area of China: A modelling study
Source: PLoS Negl Trop Dis. 2022 May 23;16(5):e0010429. doi: 10.1371/journal.pntd.0010429 (PMC9166357; doi:10.1371/journal.pntd.0010429)
Supplement: S4 File — (DOCX) [file pntd.0010429.s016.docx]

# S4 File. Calculation of costs

## A. Costs of each component of the control strategies

As presented in **S6 Table**, the costs of each component were calculated based on literatures or market research. All costs were displayed as cost per unit in 2020 US international dollars (I$).

**Supplementary Table 6.** **Costs of each aspect** **of the control strategies^*^.**

| Item | Aspect | Description | Base cost per unit (2020 US I$) | Ranges | Distribution | Reference |
| --- | --- | --- | --- | --- | --- | --- |
| **Chemotherapy** | Drug | Praziquantel（75mg/kg） | 8.12 | - | - | Market research |
|  |  | Albendazole（3.2g） | 2.39 | - | - | Market research |
|  | Delivery | Costs related to chemotherapy excluding drug costs | 0.51 | Base ± 25% | Triangular | [1] |
|  | Cost to identify infected individuals | Stool examination (Kato-Katz) | 5.15 | Base ± 25% | Triangular | [1] |
| **IEC** | - | Information education, group meetings, IEC materials and media, etc | 1.12 | Base ± 25% | Triangular | [2] |
| **Environmental modification** | Initial investment cost | Removal of toilets (per toilet) | 97.94 | Base ± 25% | Triangular | [3] |
|  | Recurrent cost | Construction of septic tank toilets (per toilet) | 452.65 | Base ± 25% | Triangular | [4] |
|  |  | Maintenance of toilets (per toilet per year) | 88.86 | Base ± 25% | Triangular | [5] |

^*^Costs were assumed to follow a triangular distribution, with the mode being the value extracted from the reference, and the upper and lower bounds being plus or minus 25% of the mode.

## B. Calculation of costs

The total costs were composed of three parts and calculated as below:

$$Total costs=({Cost}_{MDA}+{Cost}_{IEC})+{Cost}_{EM} \left( 1 \right)$$

Here, ${Cost}_{MDA}$, ${Cost}_{IEC}$, and ${Cost}_{EM}$ represent the chemotherapy costs, IEC costs, environmental modification costs, respectively. For each component, the costs each year were assumed consistent and the calculation formulas were presented as below:

$$\begin{aligned} {Cost}_{MDA}=\sum_{i=1}^{n} \sum_{g=1}^{4} N_{g,i}\times C_{m,g,i}\times\left( {Cost}_{pm,g,i}+{Cost}_{dev,g,i} \right)+I\times M_{g,i}\times C_{m,g,i}\times kk\#\left( 2 \right) \end{aligned}$$

$$\begin{aligned} {Cost}_{IEC}=\sum_{i=1}^{n} \sum_{g=1}^{4} N_{g,i}\times C_{e,g,i}\times{Cost}_{IECP,g,i}\#\left( 3 \right) \end{aligned}$$

$$\begin{aligned} {{Cost}_{EM}=N}_{toilet}\times C_{d}\times{( Cost}_{pd,ini}+\sum_{i=1}^{n} {Cost}_{pd,rec})\#\left( 4 \right) \end{aligned}$$

In above formulas, *n* indicates the number of years, *i* indicates the *i*^th^ year, and *g* represents human groups with different raw-fish-consumption behaviors. $N_{g,i}$ indicates the number of targeted populations, determined by the types of targeted population of chemotherapy in each strategy. ${Cost}_{MDA}$consisted of per capita drug price (${Cost}_{pm,g,i}$) and delivery cost (${Cost}_{dev,g,i}$). In this study, drug costs were calculated separately for each drug type (PZQ or ABZ). As suggested in previous studies, delivery costs of chemotherapy were mainly related to several categories: salaries, transportation, administrative fees, program running, community awareness activities, training and drug distribution, etc, which represented costs related to chemotherapy excluding drug costs [6,7]. $C_{m,g,i}$ indicates the coverage rate of chemotherapy. *I* represents indicator function, which is set as 1 when the targeted population of chemotherapy is positive individuals, otherwise set as 0. $M_{g,i}$ indicates the number of people accepting diagnostic examination, which is the total number of people in *g*^th^ group, and $kk$ indicates the per capita diagnostic costs. $M_{g,i}\times C_{m,g,i}\times kk$ indicates extra diagnostic costs.

IEC aims to improve health knowledge and change habits of eating raw fish. Since it is hard to change people’s raw-fish eating habits in *C. sinensis* infection endemic areas [8], we assumed that IEC should be implemented every year to have a continuous impact. Therefore, the costs were calculated as the sum of each year cost of IEC and we assumed the per capital cost of IEC consistent per year. In formula (3), $C_{e,g,i}$ and ${Cost}_{IECP,g,i}$ represent the coverage rate (the proportion of people receiving IEC among the targeted population) and cost of IEC per capita, respectively.

In formula (4), ${Cost}_{EM}$ was composed of the initial investment of a toilet (${Cost}_{pd,ini}$) and the recurrent costs (${Cost}_{pd,rec}$). $N_{toilet}$ represents the number of unimproved toilets. $C_{d}$ represents the coverage rate of sanitation toilets. For environmental modification, by assuming that one toilet was possessed per household, $N_{toilet}$ could be calculated by multiplying the number of local households by the proportion of unimproved toilets, where the number of households was derived from statistical yearbook [9] and the proportion of unimproved toilets referred to the data of Guangdong province [10]. ${Cost}_{pd,rec}$ indicates the recurrent costs per year, which is calculated as ${Cost}_{pd,rec}=\frac{{Cost}_{create}}{L_{toilet}}+{Cost}_{maintain}$. ${Cost}_{create}$ is the costs of constructing a toilet and $L_{toilet}$ is the lifetime of toilets which was set as twenty years [11,12], as the toilets are fixed assets [13]. Maintaining costs (${Cost}_{maintain}$) are the costs of septic disposal (i.e., electricity for septic disposal and emptying of septic tanks) [12]. In this study, the type of newly constructed toilets was considered as the three-compartment septic tank toilets, which are commonly used in rural areas [5].

# References

1. Qian M, Zhou C, Zhu H, Chen Y, Zhou X. Comparison on different treatment strategies against Clonorchis sinensis infection. Infect Dis Poverty. Forthcoming 2021.

2. Fang Y. [A study on the epidemiology and the control measurements of Clonorchis Sinensis in Guangdong Province] [dissertation]. Guangzhou: Sun Yat-sen University; 2006. Chinese

3. Baiduzhidao [Internet]. [How much does it cost to remove a toilet on a fish pond?] (author's tranl). [cited 2021 Oct 27]. Available from: https://zhidao.baidu.com/question/589637454610479365.html?entry=qb_uhome_tag. Chinese.

4. Miao Y, Yang Z, Zhou H. [Research on rural residents’ willingness to pay for environmental sanitation improvement and influencing factors: taking toilet improvement as an example] (author's tranl). Journal of Management World. 2012;9:89-99. Chinese

5. Huang S, Yu S, Ding H. [Investigation report on rural toilet renovation in Shandong Province] (author's tranl). Chinese Rural Science and Technology. 2018 Aug;279:75-9. Chinese.

6. Lo NC, Gurarie D, Yoon N, Coulibaly JT, Bendavid E, Andrews JR, et al. Impact and cost-effectiveness of snail control to achieve disease control targets for schistosomiasis. Proc Natl Acad Sci U S A. 2018;115(4):E584-91. http://doi.org/10.1073/pnas.1708729114

7. Hugo CT, James ET, Fiona MF, T DH, Simon JB, Roy MA. Cost-effectiveness of scaling up mass drug administration for the control of soil-transmitted helminths: a comparison of cost function and constant costs analyses. Lancet Infect Dis. 2016;16(7):838-46. http://doi.org/10.1016/S1473-3099(15)00268-6

8. Lin S, Huang J, Li H. [Analysis on the infection status of liver flukes in villages and towns of Water Network in Pearl River Delta] (author's tranl). South China J Prev Med. 2015;41(3):273-5. Chinese.

9. Zhongshan Statistical Bureau [Internet]. [Zhongshan Statistical Yearbook 2013]. [cited 2021 Oct 27]. Available from: http://stats.zs.gov.cn/tjzl/tjnj/2013nj/index.htm. Chinese.

10. Central People's Government of the People's Republic of China [Internet]. [13.49 million sanitary toilets have been built in Guangdong province] (author's tranl). [cited 2021 Oct 27]. Available from: http://www.gov.cn/xinwen/2019-10/19/content_5442178.htm. Chinese.

11. Ndeffo MM, Kjetland EF, Atkins KE, Poolman EM, Orenstein EW, Meyers LA, et al. Cost-effectiveness of a community-based intervention for reducing the transmission of Schistosoma haematobium and HIV in Africa. Proc Natl Acad Sci U S A. 2013;110(19):7952-7. http://doi.org/10.1073/pnas.1221396110

12. Hutton G, Haller L. Evaluation of the costs and benefits of water and sanitation improvements at the global level. World Health Organization.

13. Lee SH. Large scale treatment of Clonorchis sinensis infections with praziquantel under field conditions. Arzneimittelforschung. 1984;34(9B):1227-30.
