# Supplementary material for: Cost-effectiveness evaluation of different control strategies for Clonorchis sinensis infection in a high endemic area of China: A modelling study
Source: PLoS Negl Trop Dis. 2022 May 23;16(5):e0010429. doi: 10.1371/journal.pntd.0010429 (PMC9166357; doi:10.1371/journal.pntd.0010429)
Supplement: S5 File — (DOCX) [file pntd.0010429.s017.docx]

# S5 File. Calculation of DALYs

YLL and YLD per day were calculated as follows:

$$\begin{aligned} \mathrm{YLL}=\sum_{g=1}^{4} n_{d,g}\times L_{1,g}\#\left( 6 \right) \end{aligned}$$

$$\begin{aligned} \mathrm{YLD}=\sum_{g=1}^{4} n_{i,g}\times P_{h}\times L_{2,g}\times DW\#\left( 7 \right) \end{aligned}$$

For YLL calculation, the number of deaths from *C. sinensis* infection ($n_{d,g}$) was estimated as the product of the number of infections and the fatality rate of clonorchiasis [1], both of which were derived from the transmission model. $L_{1,g}$ was estimated as the life expectancy $L_{c}$ minus the average age of deaths of cholangiocarcinoma (CCA) patients $L_{d}$, where $L_{c}$ was set to be the 2015 life expectancy in China (76 year) [2]. $L_{d}$ was replaced by the age of diagnosis of CCA patients due to the difficulty of obtaining the average age of deaths from CCA and the poor prognosis of patients with CCA [3]. $L_{d}$ was assumed to follow a triangular distribution with the mode 62.6 years, and the upper and lower bounds being 51.4 and 73.8, respectively [3].

For YLD calculation, $n_{i,g}$ represents the number of incident cases. $P_{h}$ indicates the proportion of incident cases with heavy infections, thus $n_{i,g}\times P_{h}$ represents the number of disability cases. The number of incident cases $n_{i,g}$ was derived from the simulations of transmission model, expressed as $\left[ \left( 1-C_{e1,g}E_{g} \right)\beta_{h,1}+(1-C_{e2,g}E_{g})\beta_{h,g} \right]S_{h,g}I_{f}$. The proportion of incident cases with heavy infections $P_{h}$ was derived from literature: 0.0819 (95% *CI*：0.0590-0.1082) [4], and it was assumed to follow a triangular distribution with the mode being its mean and the upper and lower bounds being its 95% *CI*. The duration of disease $L_{2,g}$ was estimated as the inverse of emigration rates from infected individuals [5,6], expressed as $[\frac{1}{\mu_{h}+\mu_{d}+\gamma_{1}+\gamma_{2}}]/365$. The disability weight $DW$ was set to 0.123, namely the value adopted by WHO [7].

# References

1. Lo NC, Gurarie D, Yoon N, Coulibaly JT, Bendavid E, Andrews JR, et al. Impact and cost-effectiveness of snail control to achieve disease control targets for schistosomiasis. Proc Natl Acad Sci U S A. 2018;115(4):E584-91. http://doi.org/10.1073/pnas.1708729114

2. National Bureau of Statistics [Internet]. [Significantly enhanced its international standing and enhanced its international influence -- The 19th report on the achievements of economic and social development over the past 40 years of reform and opening up] (author's tranl). [cited 2021 Oct 27]. Available from: http://www.stats.gov.cn/ztjc/ztfx/ggkf40n/201809/t20180917_1623312.html. Chinese.

3. Kamsa-ard S, Luvira V, Suwanrungruang K, Kamsa-ard S, Luvira V, Santong C, et al. Cholangiocarcinoma trends, incidence, and relative survival in Khon Kaen, Thailand from 1989 through 2013: a population-based cancer registry study. J Epidemiol. 2019;29(5):197-204. http://doi.org/10.2188/jea.JE20180007

4. Fürst T, Keiser J, Utzinger J. Global burden of human food-borne trematodiasis: a systematic review and meta-analysis. Lancet Infect Dis. 2012;12(3):210-21. https://doi.org/10.1016/S1473-3099(11)70294-8

5. Ma Z, Zhou Y, Li J, Zhang J, Lou J, Han L, et al. [Mathematical modeling and research of infectious disease dynamics] (author's tranl). 1st ed. Beijing: Science press; 2004.

6. Hethcote HW. Three basic epidemiological models. In: Levin S.A., Hallam T.G., Gross L.J. (eds). Applied mathematical ecology. Biomathematics, vol 18. Springer, Berlin, Heidelberg. https://doi.org/10.1007/978-3-642-61317-3_5

7. World Health Organization [Internet]. WHO estimates of the global burden of foodborne diseases; c2021 [cited 2021 Oct 27]. Available from: https://www.who.int/foodsafety/publications/foodborne_disease/fergreport/en/.
