# Supplementary material for: Cost-effectiveness evaluation of different control strategies for Clonorchis sinensis infection in a high endemic area of China: A modelling study
Source: PLoS Negl Trop Dis. 2022 May 23;16(5):e0010429. doi: 10.1371/journal.pntd.0010429 (PMC9166357; doi:10.1371/journal.pntd.0010429)
Supplement: Attachment — Submitted filename: Response letter v2.docx [file pntd.0010429.s018.docx]

Guangzhou, 31 March 2022

Response to Reviewer’s Comments

Dear editors and reviewers,

We refer to our manuscript titled “Assessment of control strategies against *Clonorchis sinensis* infection based on a multi-group dynamic transmission model” (your reference No. PNTD-D-21-01633). Many thanks to you and the reviewers for the excellent comments and suggestions. We have carefully read the comments and addressed all points proposed by reviewers in the revised manuscript. Our point-to-point response were provided below. **All line numbers in the response were referred to the clean version of the revised manuscript.**

***********************

Reviewer's Responses to Questions

Key Review Criteria Required for Acceptance?

As you describe the new analyses required for acceptance, please consider the following:

**Methods**

-Are the objectives of the study clearly articulated with a clear testable hypothesis stated?

-Is the study design appropriate to address the stated objectives?

-Is the population clearly described and appropriate for the hypothesis being tested?

-Is the sample size sufficient to ensure adequate power to address the hypothesis being tested?

-Were correct statistical analysis used to support conclusions?

-Are there concerns about ethical or regulatory requirements being met?

Reviewer #1: (No Response)

Reviewer #2: ① Please describe the materials and methods more clearly and systematically.

**Reply**:

Thanks to the reviewer for taking time to review our research. As suggested by the reviewer’s comments, we have carefully adjusted the structure and content of the whole manuscript to make it clearer and more systematic.

In the methods section, original five subsections were reduced to four, currently including methods overview, simulation of interventions, cost-effectiveness analysis and uncertainty analysis. The original ‘The optimal control strategy’ subsection was simplified and integrated into the ‘cost-effectiveness analysis’ subsection, considering that the determination of optimal control strategy through ‘next best comparator’ approach was to select the strategy with best cost-effectiveness, thus was a part of the cost-effectiveness analysis (**lines 161-198 in the revised manuscript**).

In the ‘methods overview’ subsection, unnecessary description was removed or moved to the appendix. Detailed location and population information of the subjected endemic area, namely Fusha Town, was added in the revised manuscript (**lines 117-122 in the revised manuscript**). In general, as presented in this subsection, the whole analysis procedure was shown as below:

a. Adapt the previous multi-group dynamic transmission model for further cost-effectiveness analysis (**lines 115-117 in the revised manuscript**)

b. Perform parameter estimation based on Fusha Town data (**lines 117-122 in the revised manuscript**)

c. Add intervention parameters to the basic model to develop full model (**lines 125-126 in the revised manuscript, details displayed in Appendix 2A**)

d. Simulate interventions: three recommended strategies, and different combinations of control measures (**lines 126-131 in the revised manuscript**)

e. Perform cost-effectiveness analysis by estimating costs and DALYs, and obtain the optimal control strategy by ‘next best comparator’ method (**lines 131-134 in the revised manuscript**)

In the ‘simulation of interventions’ subsection, we made the content easier to understand by classifying the strategies being evaluated into two categories (**lines 139-156 in the revised manuscript**). The first category was recommended strategies, and the second category was various combinations of three commonly used measures. We also elaborated that two types of drugs (PZQ and ABZ) were selected as chemotherapy options, in order to make the analysis more comprehensive (**lines 154-155 in the revised manuscript**).

In the ‘cost-effectiveness analysis’ subsection, detailed calculation formulas were moved to the appendix. We briefly summarized the components of costs of control measures (**lines 164-170 in the revised manuscript**), and the methods of estimating YLDs and YLLs (**lines 175-180 in the revised manuscript**). The procedure of determining the optimal control strategies was simplified and integrated into this subsection (**lines 188-198 in the revised manuscript**).

In the ‘uncertainty analysis’ subsection, some sentences and words were modified to make the description more clearly (**lines 200-207 in the revised manuscript**).

② Please replace the previously well-known methods as references.

**Reply:**

As mentioned by the reviewer, some conventional methods adopted in the research were widely used in previous studies. Calculation of DALYs was a well-known method in assessing health outcomes from disease, so that we made adjustments to the description of estimating DALYs in the revised manuscript (**lines 174-190 in the revised manuscript**). Detailed formulas were removed from the main text and the convention method of estimating YLDs that we followed was cited as **references 27**. Moreover, the ‘next best comparator’ method which was also widely used in cost-effectiveness analysis was cited as **reference 13**, and the specific procedure was briefly introduced in the revised manuscript (**lines 188-198 in the revised manuscript**).

③ Please define the population characters of a subjected endemic area, Fusha Town.

**Reply:**

Thanks for the reviewer of reminding us to define the population characters of the subjected endemic area, namely Fusha town. Firstly, we designated the detailed location of Fusha town and introduced the *C. sinensis* infection rate of Fusha town in 2017 (**lines 110-112 in the revised manuscript**) which was derived from previous survey data **(reference 21)**. Then, in the ‘methods overview’ subsection, we further elaborated the coordinates, population number, eating habits and fishing condition of Fusha town (**lines 118-122 in the revised manuscript**), to more specifically define the characters of local residents. Due to the fact that life cycle of *C. sinensis* is closely related to raw-fish eating behaviour and fishing industry development, we described the characters of Fusha town related to these issues. It showed that Fusha town is a typical endemic area of *C. sinensis* infection, suggesting its appropriateness of being used to estimate the parameters.

④ Please refer the guidelines of PLOS NTD.

**Reply:**

We have carefully read the requirements for ‘Method’ section in the PLOS NTD submission guideline, and make sure that the manuscript has met the criterion. The original data, namely infection rate data of Fusha town, can be found in previous studies and was listed in the appendix (**Appendix 1B**). Moreover, we ensured that enough details of the statistical methods, including construction of the transmission model (**Appendix 1A and 2A**), parameter estimation (**Appendix 1C**), cost calculation (**Appendix 3B**), DALYs estimation (**Appendix 3C**) and uncertainty analysis (**lines 199-213 in the revised manuscript**), etc, were included in the main text and the appendix.

--------------------

**Results**
-Does the analysis presented match the analysis plan?
-Are the results clearly and completely presented?
-Are the figures (Tables, Images) of sufficient quality for clarity?

Reviewer #1: (No Response)

Reviewer #2: ① Please describe the results more clearly and systematically.
② Please refer the guidelines of PLOS NTD.

**Reply:**

As suggested by the reviewer’s comments, we have made amendments to the contents of the results section and carefully referred the submission guidelines of PLOS NTD. The guideline mentioned that ‘do not duplicate data in graphs and tables’, so that some numerical results that have been presented in the tables was deleted from the main text, including the results of WHO and China strategy (**lines 245-247 in the original manuscript**) and the total costs results (**lines 248-251 in the original manuscript**), only main results (**lines 217-222 in the revised manuscript**) were preserved. In this case, the results were presented more clearly and understandable.

Regarding the display of results of the optimal control strategies, we changed the presentation form of the numerical results. Instead of merely reporting that which strategy was the overall optimal control strategy, we presented the proportion in 500 simulations of the corresponding optimal strategy (**lines 246, 249, 260 and 264 in the revised manuscript**) to make the results more concrete. Moreover, the optimal strategies results for other targeted population of chemotherapy (i.e., whole, at-risk and positive population) were moved from main text to **supplementary tables 10 and 11**, to make this section more focused on the main results. The optimal strategies to reach infection control and transmission control were combined into one table (**table 2**).

--------------------

**Conclusions**

-Are the conclusions supported by the data presented?

-Are the limitations of analysis clearly described?

-Do the authors discuss how these data can be helpful to advance our understanding of the topic under study?

-Is public health relevance addressed?

Reviewer #1: (No Response)

Reviewer #2: Please describe the conclusion more objectively based on the findings obtained by the present study.

-> In this study, the long term cost-effectiveness for control strategies of clonorchiasis was evaluated with different coverages and combinations of control measures in a highly endemic area of China. The findings may be help to make disease prevention and control policies for clonorchiasis in highly endemic areas

**Reply:**

Thanks for the reviewer’s suggestion on how to more objectively describe the conclusion. The final paragraph was modified according to the opinion (**lines 423-428 in the revised manuscript**).

--------------------

**Editorial and Data Presentation Modifications?**

Use this section for editorial suggestions as well as relatively minor modifications of existing data that would enhance clarity. If the only modifications needed are minor and/or editorial, you may wish to recommend “Minor Revision” or “Accept”.

Reviewer #1: (No Response)

Reviewer #2: (No Response)

--------------------

**Summary and General Comments**

Use this section to provide overall comments, discuss strengths/weaknesses of the study, novelty, significance, general execution and scholarship. You may also include additional comments for the author, including concerns about dual publication, research ethics, or publication ethics. If requesting major revision, please articulate the new experiments that are needed.

Reviewer #1: (No Response)

Reviewer #2: This manuscript is on the the cost-effectiveness evaluation about three different control strategies for clonorchiasis in a highly endemic area of China. It has important informations on the cost-effectiveness in control strategies for clonorchiasis. However, it has lots of limited points to be published in PLOS NTD. First of all, this manuscript should be revised English by a native speaker. It is highly questionable whether albendazole is an effective drug for clonorchiasis chemotherapy.

**Reply:**

Thanks again for the reviewer’s suggestions and the affirmation of the results. We have carefully revised the whole manuscript based on the comments and the submission guidelines, including English grammar and phrasing, to make the description of the article more logical. Regarding the question of the effectiveness of albendazole, previous studies (**cited as reference 38**) suggested that PZQ and ABZ had similar efficacy. Here, we selected two types of drugs as chemotherapy options to make the cost-effectiveness analysis more comprehensive (**lines 348-350 in the revised manuscript**). The use of ABZ was regarded as a possibility, not an alternative, and we also agreed that PZQ was a commonly used drug in chemotherapy for clonorchiasis (**lines 365-369 in the revised manuscript**). The optimal control strategies obtained through uncertainty analysis were displayed for both PZQ and ABZ.

1. Title: Please refer the revised title.

“Evaluation of cost-effectiveness about three different control strategies for Clonorchis sinensis infection in a highly endemic area of China”or

“Cost-effectiveness evaluation about three different control strategies for clonorchiasis in a highly endemic areas of China

**Reply:**

According to the reviewer’s suggestion, the revised title was ‘Cost-effectiveness evaluation of different control strategies for *Clonorchis sinensis* infection in a high endemic area of China: a modelling study’. In addition to the three recommended strategies, the study also performed cost-effectiveness analysis of the strategies with different combination of control measures, therefore the title was revised as ‘evaluation of different control strategies’. Moreover, in order to present the study design, we added a subtitle ‘a modelling study’.

2. Abstract

① Please briefly describe the purpose of this study in the beginning.

Line 21-28: Clonorchiasis is an important food-borne parasitic disease caused by Clonorchis sinensis infection. Long-term cost-effectiveness of control strategies needs to be evaluated to provide information for establishing control programs. We performed cost effectiveness analysis of three recommended strategies (i.e., WHO, Chinese and Guangdong strategies) and different combinations of measures (i.e., preventive 26 chemotherapy, information, education, and communication (IEC) and environmental modification) under a high endemic environment, based on a multi-group transmission model of C. sinensis infection.

-> Present study was performed to evaluate the cost effectiveness on 3 different control strategies, i.e., WHO, Chinese and Guangdong, for clonorchiasis and also analized the effects of different control measures, i.e., preventive chemotherapy, information, education, and communication (IEC) and environmental renovation, in a highly endemic region in China based on a multi-group transmission model of C. sinensis infection.

**Reply:**

We have modified the paragraph of introducing the purpose of the study in the abstract, based on the reviewer’s suggestion (**lines 21-27 in the revised manuscript**).

② Please describe the materials and methods more clearly and briefly.

Line 28-30. We set the intervention duration for 10 years and post intervention period for 50 years. The optimal control strategy was obtained using next best comparator method. Uncertainty analysis was further conducted. + informations on the subjected region and disability-adjusted life years (DALYs).

**Reply:**

The information of the subjected region (i.e., Fusha town) was added to the abstract (**lines 27-28 in the revised manuscript**). The methods described in the abstract included the simulation period, the method of identifying highly cost-effective strategies, and the method of selecting the optimal control strategy, which were essential methods adopted in the study (**lines 29-34 in the revised manuscript**). According to the reviewer’s comments, we further added the information about estimating costs and DALYs to the abstract (**lines 30-31 in the revised manuscript**).

③ Please describe the results more clearly and systematically.

Line 30-42: We found that Guangdong strategy was most cost-effective among the three recommended strategies, with cost per DALY averted being $172 (95% CI: $143-$230) US for praziquantel and $106 (95% CI: $85-$143) US for albendazole. The optimal strategies with high proportions in uncertainty analysis tended to be the ones combined with preventive chemotherapy, IEC and environmental modification, under coverages of all being 100%. For praziquantel, chemotherapy of the optimal strategies for infection control (maintaining a prevalence<5%) and transmission control (maintaining a prevalence<1%) were targeted on at-risk population and positive population, respectively, with coverages 100% and costs per DALY averted $202 (95% CI: $168-40 $271) US and $339 (95% CI: $265-$453) US, respectively. For albendazole, the chemotherapy of optimal strategies was targeted on whole population, and the costs per DALY averted was $205 (95% CI: $166-$272) US.

-> In Guangdong strategy, the cost per DALY (disability-adjusted life year) avrted were $172 (95% CI: $143-$230) US for praziquantel and $106 (95% CI: $85-$143) US for albendazole, which was most cost-effective among 3 control strategies for clonorchiasis.

Please reveal the evidences (findings) as numerical results you obtained and don’t make them descriptively.

**Reply:**

Based on the reviewer’s comments, we have shortened the paragraph of presenting results in the abstract and describe the numerical results more briefly (**lines 34-41 in the revised manuscript**). The results of highly cost-effective strategies to achieve infection control were added to the abstract (**lines 37-39 in the revised manuscript**). To make the contents more readable, only the optimal strategy to reach infection control for drug PZQ was displayed in abstract (**lines 37-41 in the revised manuscript**). Results for transmission control and drug ABZ were obtained by the same approach and were displayed in the main text (**lines 41-42 in the revised manuscript**).

④ Please describe the conclusion more objectively based on the findings obtained by the present study. The conclusive remarks are too long.

Line 42-47: In this study, we evaluated the long term cost-effectiveness of control strategies with different coverages and combinations, not restricted to limited intervention types. The numerical results provided information for developing disease prevention and control policies on C. sinensis infection in high endemic areas. Moreover, the method adopted in this study is applicable for assessment of optimal strategies in other endemic areas.

-> In this study, the long term cost-effectiveness for control strategies of clonorchiasis was evaluated with different coverages and combinations of control measures in a highly endemic area of China. The findings may be help to make disease prevention and control policies for clonorchiasis in highly endemic areas.

**Reply:**

Modification of the conclusion part has been made in the revised abstract as following (**lines 42-45 in the revised manuscript**):

The findings may help to develop control policies for *C. sinensis* infection in high endemic areas. Moreover, the method adopted is applicable for assessment of optimal strategies in other endemic areas.

Part of the conclusion in the original abstract was duplicated with the purpose of the study, so that it was deleted (**lines 42-44 in the original manuscript**).

3. Introduction

① Please describe the background of this study more specifically.

Line 50: Caused by infection with Clonorchis sinensis, clonorchiasis is one of the most

-> Clonorchiasis, Clonorchis sinensis infection, is one of the

Line 55: environmental modification -> environmental renovation

Line 60: people’s healthy behaviors -> people’s behaviors

hygiene habits -> hygienic habits

**Reply:**

Thanks for the reviewer’s comments on how to more clearly present the background of this study. Corresponding wording was modified in the revised manuscript (**lines 70-71 in the revised manuscript**). We carefully considered the proper saying of ‘environmental modification’ and we found that in most related studies, this definition was referred as ‘environmental improvement’ or ‘environmental modification’. Taking into account the reviewers' suggestion and other literatures, we changed ‘environmental modification’ to **‘environmental improvement’** (**e.g. line 75 in the revised manuscript**).

In the background part, the importance and necessity of carrying out cost-effectiveness analysis of control strategies for *C. sinensis* infection, which was the main study purpose, was more specifically elaborated (**lines 84-87 in the revised manuscript**). We pointed out that integrated control programs on *C. sinensis* infection have been emphasized, leading to requirements of increasing costs and labours. Therefore, the cost-effectiveness analysis needs to be performed to determine which strategy can realize the best control effect with the lowest costs.

② Please describe the purpose elicited from backgrounds of this study in the last paragraph.

Line 91: Please more detailedly designate “Fusha Town”(location and/or province in China) and reveal the references for the clue of clonorchiasis endemic.

**Reply:**

At the beginning of the manuscript, we described the purpose of the study (**lines 105-110 in the revised manuscript**). We added the location and infection rate information of Fusha town in the introduction part (**lines 110-112 in the revised manuscript**), and other more specific information in the ‘method overview’ part (**lines 118-122 in the revised manuscript**). The source of the infection rate of Fusha Town (33.67% in 2017) was cited as **reference 21**, which was revealed as evidence of clonorchiasis endemic in Fusha town.

--------------------

**The changes we have made in the manuscript were additionally listed as below:**

**Title:**

According to the reviewer’s comment, the new title was revised as ‘Cost-effectiveness evaluation of different control strategies for *Clonorchis sinensis* infection in a high endemic area of China: a modelling study’.

**Abstract**

**(1) Lines 21-28**

Based on the reviewer’s comments, the introduction of the purpose of the study was modified, and the information of subjected endemic area (i.e., Fusha Town) was added.

**(2) Lines 30-31**

According to the reviewer’s comments, we further added the information about estimating costs and DALYs of the control strategies which is the main component of methods to the abstract.

**(3) Lines 31-33**

The method of identifying highly cost-effective strategies was added in the abstract.

**(4) Lines 34-42**

The results displayed in the abstract were shortened and simplified. We added the results of highly cost-effective strategies obtained by corresponding methods. For optimal strategies, only the results for infection control and for drug PZQ were displayed in the abstract. Similar results for transmission control and for drug ABZ were obtained and shown in the main text.

**(4) Line 42-45**

The conclusion part was revised according to the reviewer’s comment. The contents which were duplicated with the study purpose were deleted.

**Introduction**

**(1) Lines 70-71**

Some description in the manuscript was revised based on the reviewer’s comments.

**(2) Line 75**

Environmental modification -> environmental improvement.

The other ‘environmental modification’ phrases in the manuscript have also been modified in the same way.

**(3) Line 80**

people’s healthy behaviors -> people’s behaviors

hygiene habits -> hygienic habits

**(4) Lines 84-87**

Considering that cost-effectiveness analysis was the main purpose, we further elaborated the importance and necessity of carrying out cost-effectiveness analysis of control strategies for *C. sinensis* infection. As presented in the manuscript, integrated control programs on *C. sinensis* infection have been emphasized in recent years, leading to requirements of increasing costs and labours. Therefore, the cost-effectiveness analysis needs to be performed to determine which strategy can realize the best control effect with the lowest costs.

**(5) Lines 89-104**

The orders of introducing the current research status were modified. Firstly, we discussed that previous studies which performed cost-effectiveness analysis of *C. sinensis* control strategies did not use DALYs proposed by WHO as indicators. Secondly, we pointed out that many studies were carried out from practical perspective instead of modelling approach. Then we discussed the advantages of modelling approach, leading to the study purpose presented in the next paragraph.

**(6) Lines 107-110**

We summarized the control strategies being evaluated in the manuscript, including three recommended strategies and combination of common measures.

**(7) Lines 110-112**

We pointed out that Fusha town was used as the study area in this study. The location and infection rate of Fusha town was added.

**Methods**

**(1) Lines 115-117**

The description of incorporating disease fatality parameter to the basic model was moved to the appendix.

**(2) Lines 118-122**

Detailed location and population information of the subjected endemic area, namely Fusha Town, was added in the revised manuscript.

**(3) Lines 114-134**

The method overview subsection was organized and presented based on procedures below:

a. Adapt the previous multi-group dynamic transmission model for further cost-effectiveness analysis

b. Perform parameter estimation based on Fusha Town data

c. Add intervention parameters to the basic model to develop full model

d. Simulate interventions: three recommended strategies, and different combinations of control measures

e. Perform cost-effectiveness analysis by estimating costs and DALYs, and obtain the optimal control strategy by ‘next best comparator’ method

**(2) Lines 125-126**

To make the manuscript more systematic, the description of establishing a full model with interventions based on the modification of the basic model was moved to the ‘method overview’ subsection.

**(3) Lines 139-156**

We classified the strategies being evaluated into two categories. The first category was recommended strategies, and the second category was various combinations of three commonly used measures. We also described the specific combinations of measures adopted by three recommended strategies.

**(4) Lines 154-155**

We elaborated that PZQ and ABZ were both selected as chemotherapy options without preference, in order to make the analysis more comprehensive.

**(5) Lines 162-164**

We summarized the overall method of performing cost-effectiveness, in reference to procedures of other studies.

**(6) Lines 164-170, and lines 175-180**

In order to make the contents more understandable, the components of costs of control measures, and the general methods of estimating YLDs and YLLs were briefly introduced in the main text.

**(7) Lines 182-183**

We pointed out that ICERs were used as indicators to evaluate cost-effectiveness of control strategies.

**(8) Lines 185-188**

We modified the description of identifying highly cost-effective strategies.

**(9) Line 188-198**

The original subsection ‘the optimal control strategy’ was integrated to the ‘cost-effectiveness analysis’ subsection, and procedure of determining the optimal control strategies was simplified. The next best comparator method used to obtain the optimal control strategy was cited as **reference 13**.

**(10) Line 200-207**

Some sentences and words were modified to make the description more clearly.

**Results**

**(1) Line 215**

Three recommended strategies -> The three recommended strategies

**(2) Lines 217-222**

Following the PLOS NTD submission guideline, the numerical results of amount of DALYs averted were removed from the main text. Instead, we described that which strategy averted the highest DALYs among three recommended strategies. The numerical results of the total costs were also removed, because they were not the main results.

**(3) Line 223**

Costs per DALY averted -> ICER

The indicator of cost-effectiveness of each control strategy was modified as ‘ICER’.

**(4) Lines 227-229**

We pointed out that the WHO strategy has the lowest cost-effectiveness.

**(5) Lines 234**

Effective -> highly cost-effective. This modification was in accordance with the method section.

**(6) Lines 239-241**

The results displayed in appendix were moved to the ending of this subsection.

**(7) Line 252**

Costs per DALY averted -> ICER

The indicator of cost-effectiveness of each control strategy was modified as ‘ICER’.

**(8) Lines 242-270**

We changed the presentation form of the numerical results of determining optimal control strategies. In the revised manuscript, we reported that the proportion of the control strategy being optimal in 500 simulations. For example, when the chemotherapy drug was PZQ, the chemotherapy targeted on at-risk population combined with IEC and environmental modification with coverage all being 100% was the optimal strategy in 48.0% of simulations. When the drug was ABZ, the chemotherapy targeted on whole population combined with IEC and environmental modification with coverage all being 100% was the optimal strategy in 84.6% of simulations. In this case, more concrete results were provided.

**(10) Lines 268-270**

The optimal strategies for other types of targeted population of chemotherapy were removed from the main text and were displayed in the supplementary tables 10 and 11. In this case, we summarized only main results in the main text.

**(11) Lines 272-276**

The optimal cost-effective strategies for any targeted population of chemotherapy to achieve infection control and transmission control were originally separated. In the revised manuscript, they were combined into one table (**table 2**). Moreover, the results considering the analysis for other targeted population were removed in **table 2**, so that the display became clearer.

**Discussion**

**(1) Lines 348-350**

We added previous evidence which suggested that PZQ and ABZ had similar efficacy, to explain the reason of selecting these two drugs as chemotherapy options. As presented in the revised manuscript, the purpose was to make the cost-effectiveness analysis more comprehensive.

**(2) Line 351**

We added the word ‘namely’ to refer PZQ as the more expensive drug.

**(2) Line 365-371**

We made deeper discussion about the two chemotherapy drugs. PZQ was widely used in practice and ABZ was more acceptable with lesser smell and taste. Without preference of these two drugs, we suggested that the choice of drug type should follow local conditions.

**Conclusion**

**Lines 423-428**

Based on the reviewer’s comment, we modified the description of conclusion to make it more objective.

**References**

The order of the references has been modified according to the adjustment of the main text. **Reference 21** was newly added to refer to the infection rate of *C. sinensis* in Fusha town. **Reference 27** was added to cite the common methods of estimating YLDs used by other studies. **Reference 38** was added to show that studies have suggested that PZQ and ABZ had similar efficacy.

**Supporting information**

S10 and S11 tables were added.
